# Sulfonic Ion‐Exchange Resins as Versatile Tools for the Oxidative Degradation of Chemical and Biological Hazardous Agents

**DOI:** 10.1002/cssc.71017

**Published:** 2026-08-26

**Authors:** Stefano Econdi, Alessandro Caselli, Simona Tomaselli, Laura Ragona, Davide Mileto, Matteo Guidotti

**Affiliations:** ^1^ CNR‐SCITEC Institute of Chemical Sciences and Technologies “Giulio Natta” Milan Italy; ^2^ Department of Chemistry University of Milan Milan Italy; ^3^ Laboratory of Clinical Microbiology Virology and Bioemergency Hospital “L. Sacco” Milan Italy

**Keywords:** chemical hazardous agents, decontamination, hydrogen peroxide, ion‐exchange resins, viruses and bacteria

## Abstract

Commercial sulfonic styrene–divinylbenzene ion‐exchange resins are activated with aqueous H_2_O_2_ to generate metal‐free decontamination systems that combine strong Brønsted acidity with immobilized oxidizing capability. Among five tested materials, Amberlyst 15 dry showed the best performance in terms of oxidant immobilization capacity and promoting the oxidative degradation of the sulfur mustard simulant (2‐chloroethyl)ethyl sulfide, CEES, and the organophosphorus pesticide malathion under very mild conditions. Control experiments with K_2_CO_3_‐exchanged resin demonstrate that efficient decontamination requires the synergy between surface acidity and peroxide functionality. The activated resins also display rapid biocidal activity, strongly reducing viable *Escherichia coli* and *Staphylococcus aureus* and completely suppressing the infectivity of HSV‐1 and SARS‐CoV‐2 within min. These findings identify peroxide‐activated sulfonic resins as simple, sustainable, regenerable, and versatile tools for efficient combined hazardous chemical and biological decontamination.

## Graphical abstract

Hydrogen peroxide (H_2_O_2_) activation endows sulfonic ion‐exchange resins with synergistic Brønsted acidity and surface‐immobilized oxidants. Activated Amberlyst 15 promotes degradation of 2‐chloroethyl ethyl sulfide (2‐CEES) and malathion while rapidly inactivating *Escherichia coli* (*E. coli*), *Staphylococcus aureus* (*S. aureus*), herpes simplex virus type 1 (HSV‐1) and severe acute respiratory syndrome coronavirus 2 (SARS‐CoV‐2).

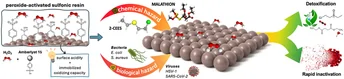

1

The rapid and reliable abatement of highly toxic chemical and biological agents remains a critical challenge for global security. The tailored design of surface functional groups in heterogeneous catalysts represents a powerful strategy for developing solid materials capable of safe and sustainable decontamination of hazardous contaminants [1, 2]. Nanostructured inorganic oxides, micro‐ or mesoporous molecular sieves, and metal–organic frameworks have demonstrated excellent performance in the adsorption and catalytic degradation of hazardous chemical agents at laboratory scale [3, 4, 5]. These systems have then reached, in some successful cases, technological maturity for deployment in prototypes, fabrics, or commercially available decontamination systems [6, 7, 8, 9, 10].

Among polymer‐based materials, copolymer resins functionalized with either metal complexes or tailored high‐surface area sorbents were integrated, in the past decades, in the formulation of military‐grade personal decontamination kits for the rapid removal and detoxification of chemical warfare agents and biological pathogens from exposed body parts and protective garments (e.g*.*, the M291 resin kit for NATO armed forces) [11, 12, 13, 14]. However, to the best of our knowledge, no systematic studies have so far investigated how simple, commercially available ion‐exchange resins can be activated and applied to the decontamination of chemical and biological highly hazardous agents. These materials therefore represent a versatile, metal‐free, and cost‐effective tool for the in situ degradation of highly hazardous sulfur‐ and phosphorus‐containing chemicals, as well as for the inactivation of pathogenic viruses and bacteria.

Herein, we report a strategy to exploit arenesulfonic functional groups at the surface of acid cation‐exchange resins by preactivating them with aqueous hydrogen peroxide. In this way, the strongly acidic sulfonic moieties are converted into active sites combining pronounced Brønsted acidity with peroxide‐based oxidizing capability. Thanks to this approach, the degradation activity is enabled by the immobilization and activation of H_2_O_2_ at the polymer surface. A comparable reactivity has previously been exploited, for chemical synthetic purposes only, in the catalytic dihydroxylation of alkenes [15] and in the oxidation of sulfides, tertiary amines, and secondary alcohols with H_2_O_2_ [16], under metal‐free conditions using perfluorinated sulfonic acid resins or polystyrene‐supported sulfonic acids. However, the use of a nonfluorinated resin, such as styrene‐divinylbenzene polymers, is a more sustainable option in terms of environmental impact and cost of the polymer itself [17, 18]. Furthermore, considering that hydrogen peroxide in a Brønsted acidic medium has shown a marked biocidal activity in the absence of metal catalytic centers [19, 20, 21], the aim of the present work is to exploit this synergistic effect for an unprecedented combined chemical and biological decontamination action.

First, the capability of sulfonic ion‐exchange resins to store and immobilize oxidant species was evaluated over five commercially available resins, all belonging to the styrene‐divinylbenzene (DVB) sulfonated copolymer family, namely, Amberlyst 15 dry, Amberlyst 16 wet, Amberlyst 36 wet, AmberLite IRC120 H, and Dowex 50WX8 200. In the tests, a weighed amount of the resin beads was activated by putting them in contact with 30 wt% H_2_O_2_ aqueous solution and dried at room temperature. Then, the content of oxidizing species in them was determined by volumetric iodometric and ceriometric titrations (see Section S1).

Both titration methods confirmed the ability of all five resins to store a remarkable amount of oxidant species (5.6–6.3 mmol H_2_O_2_/g resin; Table S1 and Figure S2). These data are in line with the average values of ion‐exchange capacity of the resins, suggesting that the amount of oxidant is comparable to the content of sulfonate moieties on the polymers. A loss of oxidant content of 50% max. was recorded after 24 h from the activation, leaving the dry resin beads at room temperature, showing an acceptable stability of the immobilized species onto the copolymer over time.

To further investigate the nature of the species present on the resin after H_2_O_2_ treatment, the results from ceriometric titrations were compared with the ones from iodometric tests. In fact, while iodometry quantifies all oxidant species present, that is, physisorbed H_2_O_2_, chemisorbed H_2_O_2_, via hydrogen bonds including bridging interactions, free‐radical species, and peroxosulfonic groups (Scheme S1) [22], Ce(IV) titration measures the reductant capability due to H_2_O_2_ only. The small, but nonnegligible, difference between the two titrations showed that about 4% of the oxidant capability is consistent with the presence of species other than free H_2_O_2_ and which are reasonably attributed to in situ‐formed peroxosulfonic groups [15]. Then, the remaining fraction (ca. 96%) is due to H_2_O_2_, immobilized within the resin structure, either through chemisorption or physisorption. Additional insights on the nature of the interaction of H_2_O_2_ with the resin were drawn from NMR studies that showed a delayed release of the oxidizing species from the activated system into liquid phase, thus suggesting nonlabile chemisorption‐like interactions with the arenesulfonic polymer's backbone (Section S1 and Figures S3, S4).

The five tested sulfonic resins were then evaluated in the catalytic degradation of (2‐chloroethyl)ethyl sulfide, CEES (Scheme 1), simulant of the CWA blister agent 2,2′‐chloroethyl sulfide (sulfur mustard, HD), and diethyl malathion, a widely used organophosphate insecticide and simulant of second‐generation sulfur‐containing nerve agents [23, 24] (Table S4). The tests were performed in liquid phase (ethyl acetate), batch reactor, and under room temperature and pressure. AcOEt was selected as the most promising reaction medium after a screening of polar protic and aprotic solvents (Section S1 and Figure S5).

**SCHEME 1:**
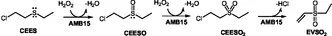
Oxidative degradation of CEES to the corresponding sulfoxide (CEESO) and further reaction to sulfone (CEESO_2_) and ethylvinyl sulfone (EVSO_2_) over activated AMB15.

The oxidative decontamination of the sulfide CEES by H_2_O_2_ leads first to the corresponding sulfoxide, CEESO, and, subsequently, to the sulfone, CEESO_2_ (Scheme 1).

All solids led to complete conversion for long reaction times (24 h), but at initial stage (0–3 h), Amberlyst 15 dry and, to a minor extent, Amberlyst 36 wet showed the best activity (Table S2).

Amberlyst 15 dry (AMB15) was therefore selected as the unique solid decontamination system for the next tests, thanks to its high oxidant immobilization capability (Figure S2), promising oxidative degradation activity, cost‐effectiveness (compared to other commercial sulfonic resins), and broad availability on the market. It is worth noting that AMB15 resin without any H_2_O_2_ pretreatment and, conversely, aq. H_2_O_2_ alone, in the absence of resin, gave rise to negligible CEES degradation (Table S2; lines 7–8).

To better understand the characteristics leading to such good performance, a targeted set of tests was carried out to differentiate the contribution due to immobilized oxidant species from the one due to resin's acidity. By exchanging AMB15 with aq. K_2_CO_3_, to obtain AMB15‐K, protonic sites were neutralized, and resin's strong Brønsted acidity was suppressed (Table 1, left). AMB15‐K, even in the presence of immobilized H_2_O_2_ but in the absence of acid sites, showed practically no CEES abatement in 24 h, thus confirming the necessary synergistic role of acidity and oxidant capability for this reaction.

**TABLE 1:** Conversion versus time of CEES and malathion on AMB15 and AMB15‐K.

%CEES conversion	15 min	1 h	5 h	24 h	%MALATHION conversion	15 min	1 h	5 h	24 h
AMB15 + H_2_O_2_	20.6	51.2	99.7	99.9	AMB15 + H_2_O_2_	23.2	36.1	63.3	99.9
AMB15‐K + H_2_O_2_	0	2.9	2.9	4.4	AMB15‐K + H_2_O_2_	3.9	4.3	5.6	6.0
AMB15 blank	0	0	1.5	7.0	AMB15 blank	1.45	1.9	2.2	2.9
H_2_O_2_ blank	0	0.5	0.5	0.9	H_2_O_2_ blank	5.3	5.6	5.9	6.4

*Note*: Conditions: 1745 ppm CEES; 220 ppm malathion in AcOEt, 298 K, 1 bar, 150 mg resin; when present, 1:5 CEES:H_2_O_2_ or 1:110 malathion:H_2_O_2_ molar ratio.

The oxidation of sulfides with H_2_O_2_ occurs in successive stages (Scheme 1), with the sulfoxide‐to‐sulfone ratio primarily dependent on reaction time [25]. It is worth noting that the sulfoxide derivative of sulfur mustard HD exhibits significantly reduced toxicity with respect to the pristine CWA, whereas the corresponding sulfone remains nearly as toxic as the original chemical warfare agent [26]. Therefore, in this case, achieving high selectivity toward CEESO is crucial for an effective decontamination.

In the first 3 h of reaction, CEES conversion reached 90%, with product distribution almost entirely favoring CEESO (Figure 1). Once the sulfide was nearly depleted, further oxidation of CEESO to CEESO_2_ began. After prolonged reaction times (up to 24 h), the formation of the hazardous sulfone became predominant. In the final stage of the reaction, the formation of ethyl vinyl sulfone (EVSO_2_) is detected too due to acid‐catalyzed elimination of HCl from the chloroethyl moiety. Interestingly, the presence of hydrolyzed compounds (e.g., 2‐(ethylsulfonyl)ethanol) and byproducts containing polymerized sulfur was ruled out through ^1^H‐ and ^13^C‐NMR analysis (Figures S7–S14) [27]. In this case, a reaction time of ca. 3 h provides an optimal compromise between high CEES abatement and selective formation of the less toxic sulfoxide, minimizing further oxidation to sulfone. By finely tuning resin's activity, with longer reaction times and a lower H_2_O_2_ concentration (6 wt%) during the activation step, CEESO was recorded as the main product with minimal amounts of CEESO_2_ (Figure S15). This is particularly significant as many conventional abatement methods often lead to the formation of substantial amounts of side products, which can be just as toxic and hazardous as the original organosulfur blister agent.

**FIGURE 1:**
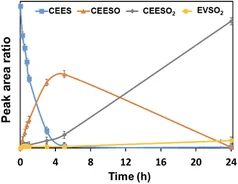
CEES degradation reaction profile over AMB15 and abundance of products formed (compound‐to‐internal std peak area ratio). Conditions: 1745 ppm CEES in AcOEt, 298 K, 1 bar, 150 mg resin; 1:5 CEES:H_2_O_2_ molar ratio; products monitored by GC‐FID.

The possibility of regenerating AMB15 was investigated in further CEES abatement cycles. The spent resin was simply recovered, rinsed with ultrapure H_2_O and AcOEt, and reactivated with aq. H_2_O_2_ and added to a fresh reaction batch. AMB15 retained its activity over four cycles, attaining more than 90% conversion in 3 h and complete CEES conversion after 5 h (Figure S16). The decrease in sulfur content (from CHNS elemental analysis, Table S3) from 12.8 to 9.9 wt% in fresh and spent AMB15 resin (after 4 runs), respectively, suggests a partial loss of the sulfonic groups along the tests and a minor, although nonnegligible degradation of the resin structure itself. In further tests, new aliquots of fresh CEES substrate were added directly into the reaction medium after 24 h, without any intermediate H_2_O_2_ treatment of the resin. The system was able to fully degrade the sulfide up to 4 subsequent times (Figure S17), thus showing a high exploitation of the resin‐immobilized oxidant over a long time under batch conditions.

When the activated AMB15 was applied to malathion, the conversion of the organophosphorus pesticide was complete within 24 h (Table 1 and Figure S18), along with the formation of various degradation products. Indeed, malathion is converted to malaoxon through oxidative desulfuration of the phosphorothioate moiety, transforming P=S to the oxon P=O analog (Figures S19 and S20). During the tests, malaoxon was detected only in small amounts and then disappeared after 24 h of the reaction. Conversely, a noteworthy presence of diethyl succinate and diethyl fumarate was observed throughout the tests, suggesting that the resin promotes the acid‐catalyzed cleavage of the P—S bond and the formation of the nontoxic dicarboxylic ester products (Table S4, Figures S21 and S22) [28, 29]. Over this substrate too, the synergistic effect between the surface acid sites and the oxidant species is essential to have an effective degradation of the nerve CWA simulant, as shown by the control tests over the nonacidic AMB15‐K resin or in the absence of H_2_O_2_ (Table 1 and Figure S18).

Aiming at developing a simple and versatile decontamination tool not only against highly hazardous chemicals but also against biological pathogens, the antibacterial and virucidal capability of activated AMB15 resin was assessed over *E. coli*, *S. aureus*, Herpes Simplex Virus type 1 (HSV‐1) and Severe Acute Respiratory Syndrome Coronavirus 2 (SARS‐CoV‐2) as target microorganisms. These are benchmark pathogens for Gram‐negative and Gram‐positive bacteria, double‐stranded DNA, and simple‐stranded RNA viruses, respectively.

The bactericidal efficacy was tested in aqueous buffer solutions, simulating real‐life biological body fluid conditions.

H_2_O_2_‐activated AMB15 resin led to fast and strong biocidal activity against *E. coli*, with a substantial 7‐log reduction in CFU after only 1 min of contact time (Figure 2A). The effect was stronger for longer times and relevant even in the presence of denser cultures (Figures 2 and S23). Untreated resin, AMB15, still exhibited a significant bactericidal activity after 5 min [30], that can be attributed to its intrinsic Brønsted acidity (Figure 2A) [31]. Conversely, K_2_CO_3_‐neutralized resin (AMB15‐K), with no prior H_2_O_2_ treatment, displayed practically no antibacterial activity, whereas H_2_O_2_‐activated AMB15‐K gave rise to a nonnegligible bacterial abatement, although lower than in the presence of AMB15 + H_2_O_2_ (Figure 2B). Altogether, these data confirm the relevant synergistic role of Brønsted acidity and immobilized oxidizing species.

**FIGURE 2:**
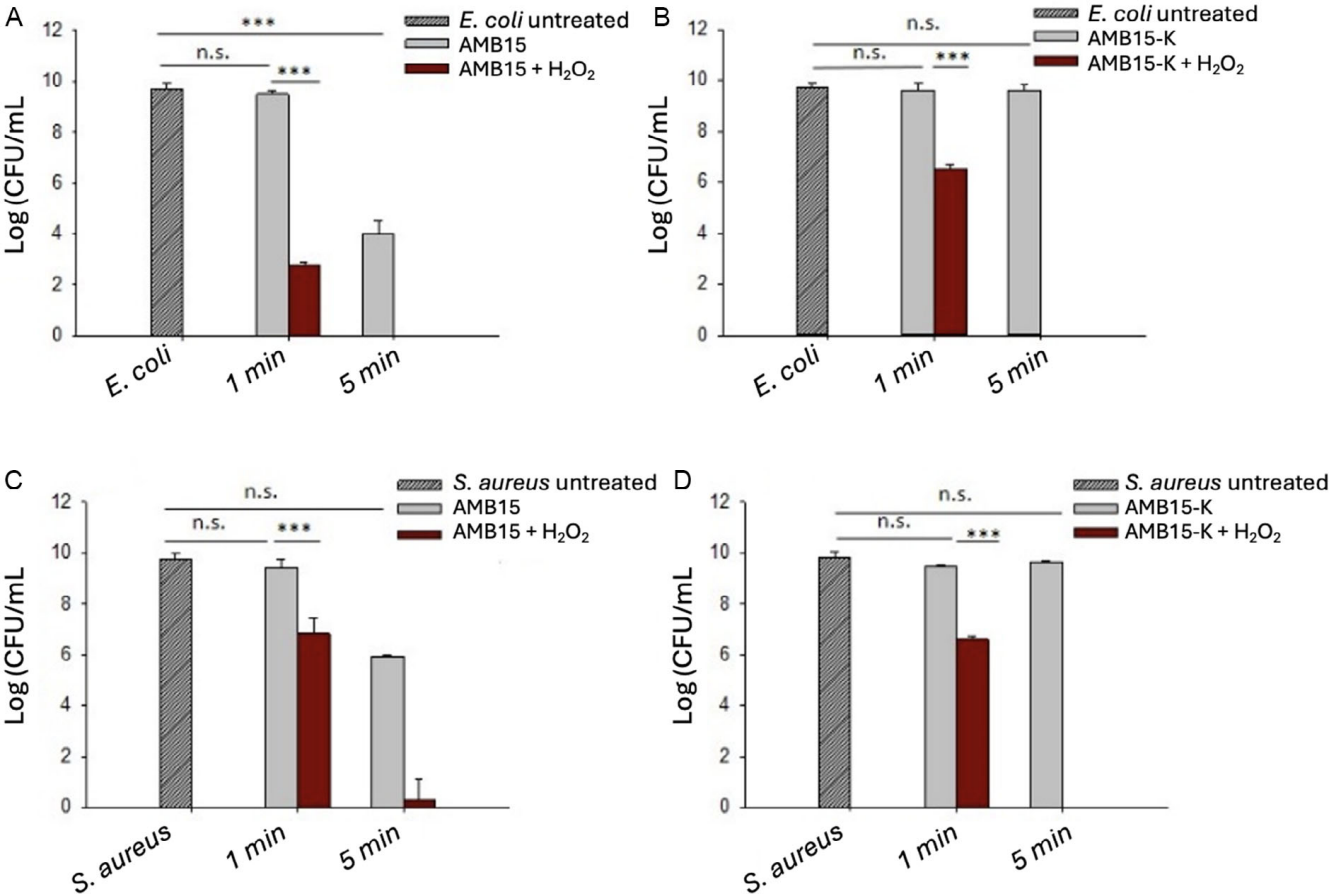
Antibacterial activity of resins against *E. coli* and *S. aureus*. *E. coli* (A,B) and *S. aureus* (C,D) cell viability measured for bacteria treated at different contact times over activated, nonactivated and neutralized AMB15 resins. Differences between treated samples and untreated *E. coli* (UNT.) or *S. aureus* were evaluated using two‐tailed Student's t‐test; ns, *p* > 0.05; *, *p* < 0.05 (not present); **, *p* < 0.01 (not present); ***, *p* < 0.001.

A similar effect was observed on *S. aureus* (Figure 2C,D). Although the biocidal activity of the resins was slightly lower than that observed for *E. coli*, activated AMB15 still showed excellent performance, with a decrease of 2 or 8 log_10_ units after 1 and 5 min of contact time, respectively. In this case too, untreated AMB15 resin succeeded in abating *S. aureus* by 4 log_10_ units after 5 min contact time due to its intrinsic acidic character.

H_2_O_2_ activation of AMB15 thus confers a relevant biocidal activity against both Gram− and Gram+ model systems after just 1 min of resin contact time.

To extend the study to other biologically hazardous agents of human relevance, the virucidal activity of the activated resins was evaluated by assessing their ability to suppress the infectivity of HSV‐1 and SARS‐CoV‐2 in Vero E6 cells (Section S1 and Figure S1). With respect to control experiments, complete inhibition of HSV‐1 replication occurred after 2 min of contact with both AMB15 and AMB15 + H_2_O_2_ at an initial HSV‐1 titer of 4 × 10^13^ PFU mL^−1^. By contrast, AMB15‐K + H_2_O_2_ showed lower activity, with a reduction of approximately four orders of magnitude, corresponding to a residual viral titer of 1.3 × 10^9^ PFU mL^−1^ (Figure 3). A similar trend was observed for SARS‐CoV‐2, for which complete loss of infectivity after 2 min was obtained with AMB15 and AMB15 + H_2_O_2_ at an initial SARS‐CoV‐2 titer of 10^6.7^ TCID_50_ mL^−1^, whereas AMB15‐K + H_2_O_2_ induced a reduction of about two orders of magnitude with a residual viral titer of 10^4.9^ TCID_50_ mL^−1^. Notably, over these two target viruses, the intrinsic acidity of H_2_O_2_‐free AMB15 displayed a full suppression of viral infectivity, as observed in other H^+^‐containing sulfonic resins [32].

**FIGURE 3:**
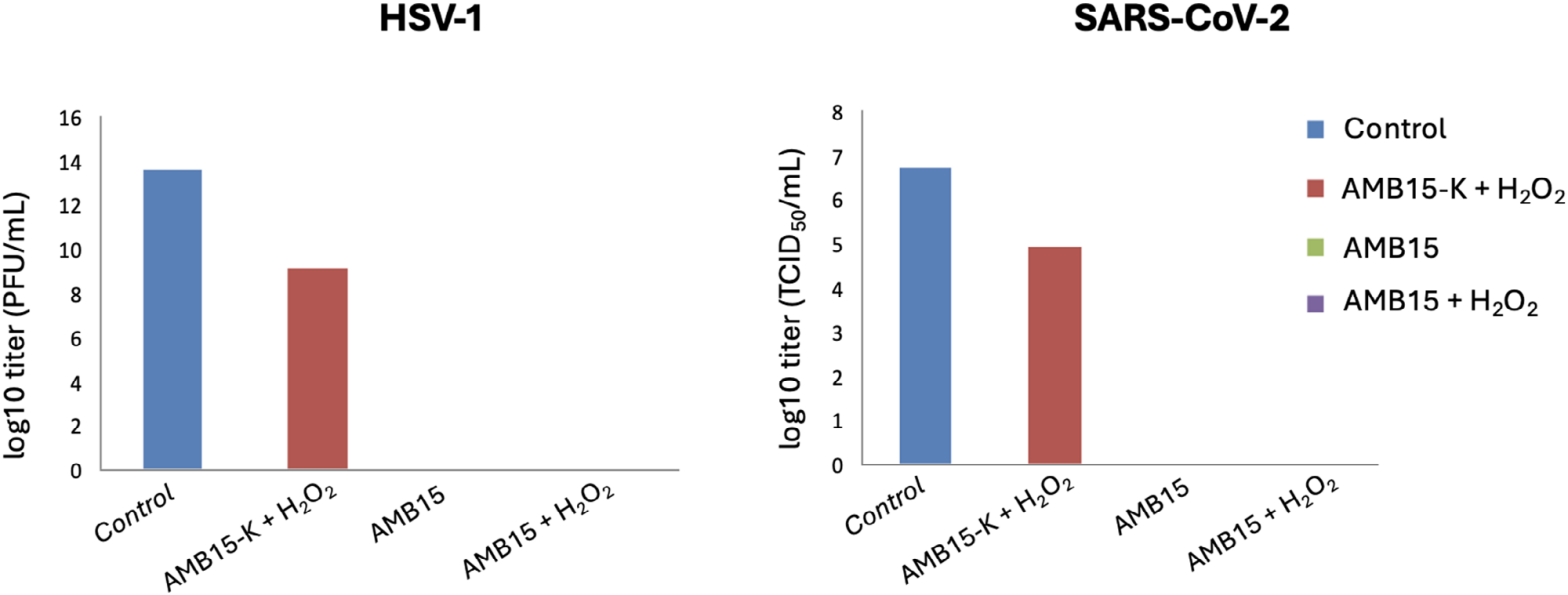
Virucidal activity of resins against HSV‐1 and SARS‐CoV‐2. Viral titers of HSV‐1 (PFU mL^−1^) and SARS‐CoV‐2 (TCID_50_ mL^−1^) measured after 2 min contact with treated and nontreated resins are shown in logarithmic scale. Initial viral titers are reported for comparison.

The lower activity observed for the system that still contains oxidizing agents but devoid of acid functionalities (AMB15‐K + H_2_O_2_) confirms that the virucidal performance arises from the combined presence of surface acidity and peroxide species.

In summary, these sets of experiments show that the activation with aqueous H_2_O_2_ of arenesulfonic‐based acid cation‐exchange resins converts them into a versatile and effective tool with excellent joint chemical and biological decontamination action. The combined presence of surface acidity and peroxide species play a crucial role in the decontamination of sulfur‐ and phosphorus‐containing highly hazardous toxic compounds as well as of bacterial and viral pathogens, highlighting the advantage of immobilizing these synergistic properties within a single integrated system. The affordability and broad‐spectrum activity of these solids make them promising candidates for practical, integrated chemical/biological decontamination technologies.

## Funding

This study was supported by Consiglio Nazionale delle Ricerche (progetto@CNR2020,SAC.AD002.173.032‐PerBiocid).

## Conflicts of Interest

The authors declare no conflicts of interest.

## Supporting information

The authors have cited additional references within the Supporting Information [33, 34, 35, 36].

## Data Availability

The data that support the findings of this study are available from the corresponding author upon reasonable request.
