# Supplementary material for: Sulfonic Ion‐Exchange Resins as Versatile Tools for the Oxidative Degradation of Chemical and Biological Hazardous Agents
Source: ChemSusChem. 2026 Aug 26;19(17):e71017. doi: 10.1002/cssc.71017 (PMC13519368; doi:10.1002/cssc.71017)
Supplement: Supplementary file 1 [file CSSC-19-e71017-s001.pdf]

Sulfonic ion-exchange resins as versatile tools for the oxidative degradation of chemical and biological hazardous agents

Stefano Econdi,[a,b] Alessandro Caselli,[a,b] Simona Tomaselli,[c] Laura Ragona,[c] Davide Mileto,[d] Matteo Guidotti*[a]

[a] S. Econdi, A. Caselli, D. Mileto, M. Guidotti
CNR-SCITEC, Institute of Chemical Sciences and Technologies "Giulio Natta"
via C. Golgi 19, 20133 Milan, Italy
E-mail: matteo.guidotti@scitec.cnr.it

[b] A. Caselli
Department of Chemistry, University of Milan
via C. Golgi 19, 20133 Milan, Italy

[c] S. Tomaselli, L. Ragona
CNR-SCITEC, Institute of Chemical Sciences and Technologies "Giulio Natta"
via A. Corti 12, 20133 Milan, Italy

[d] D. Mileto
Lab. of Clinical Microbiology, Virology and Bioemergency, Hospital "L. Sacco",
via G. B. Grassi 74, 20157 Milan, Italy

SUPPORTING INFORMATION

1. Materials and Methods

Amberlyst® 15 dry, Amberlyst® 16 wet, Amberlyst® 36 wet, AmberLite™ IRC120 H, Dowex™ 50WX8 200 were obtained from commercial vendors and used as received. Ethyl acetate (AcOEt, Sigma-Aldrich/Merck, ≥99.5% - GC grade), dichloromethane (DCM, >99.5%, D65100), ultrapure water (Merck MilliQ Academic), *n*-dodecane (GC, internal standard; Sigma-Aldrich; 297879) potassium iodide (Fluorochem, product no. 319032), sodium thiosulfate pentahydrate (Fluka, product no. 72050), cerium(IV) sulfate (Sigma-Aldrich, product no. 359009) and potassium carbonate (Sigma-Aldrich, product no. 60108) were stored at room temperature and used without further purification unless stated otherwise. Prior to use, hydrogen peroxide (Sigma-Aldrich/Merck, aq. 30 wt%), (2-chloroethyl)ethyl sulfide, CEES (TCI - C0938, >97.0% - GC grade) and diethyl malathion (PESTANAL®, analytical standard, 99.2%, 36143) were stored in a refrigerator at 277 K.
Bacterial strains, *E. coli* (ATCC 11229) and *S. aureus* (ATCC 6538), were purchased from Biogenetics Diagnostics srl. Peptone, yeast agar and BHI (Brain Heart Infusion)/BHI agar, used for *E. coli* and *S. aureus*, respectively, were purchased from Merck.

1.1. Evaluation of the oxidant capacity of resins **-** A 500 mg aliquot of each resin was placed in contact with 2.5 mL of a 30 wt.% H_2O_2 aqueous solution in a glass flask under stirring for 10 min. After this, the resin was filtered to remove excess H_2O_2 and then dried at room temperature for 3 h. Subsequently, either iodometric or cerimetric titrations were performed on the treated resins to determine the oxidant capacity.

Iodometric titration - An aliquot of resin (approx. 100-200 mg) was added to a 2% aqueous solution of KI, acidified with H_2SO_4 (5% vol/vol) (Sigma-Aldrich), and titrated with

a 0.1 M $Na_2S_2O_3 \cdot 5\ H_2O$ solution. To determine the oxidising species formed, the following formula was used:

$$\frac{mmol_{H_2O_2}}{g_{resin}} = \frac{V_{titrant} \cdot 1.7008\ (mg) / 34.0147\ \left(\frac{g}{mol}\right)}{m_{resin}\ (g)}$$

Cerimetric titration – An aliquot of pre-activated resin was titrated with a 0.05 M solution of $Ce(SO_4)_2$, as the titrant, and iron(II)–1,10-phenanthroline complex (ferroin) as an indicator, in the presence of H_2SO_4. The flask containing 150 ml of H_2SO_4 5% vol/vol, 2-3 drops of ferroin and the sample (approximately 100-200 mg) to be titrated with $Ce(SO_4)_2$, is placed in an ice bath to keep the temperature below 10 °C during titration. When the endpoint is reached, the solution changes from slightly red to blue. The content of hydrogen peroxide was determined according to the following formula:

$$\frac{mmol_{H_2O_2}}{g_{resin}} = \frac{\frac{M_{titrant}}{2}\left(\frac{mmol}{ml}\right) \cdot V_{titrant}\ (ml)}{m_{resin}\ (g)}$$

1.2. NMR study on H_2O_2 interaction with AMB15 resin – Samples of H_2O_2 at different concentrations were prepared by adding proper amount of 30 wt.% H_2O_2 aqueous solution to 600 μL DMSO-d6.

NMR samples used to follow H_2O_2 release from resin were prepared by introducing 10.8 mg of activated resin directly in the NMR tube and suspended in 600 μL of DMSO-d6.

For H_2O_2 resin activation, 200 mg of resin were immersed in 2 mL of H_2O_2 and gently stirred for 10 min on a tilting plate. The aqueous solution was then manually removed and the resins were dried using a flow of air filtered through a 0.22 μm filter.

1D ^{1}H NMR experiments were acquired at 25 °C 500 MHz (Bruker Avance III), equipped with a TXI probe. Spectra were processed and automatically integrated with Topspin 3.6 software. Concentration of H_2O_2 released from resin was estimated with respect to residual DMSO-H resonance (with known concentration).

The release of H_2O_2 from AMB15 was followed by recording a series of 1D ^{1}H-NMR spectra over time on a 10.8 mg AMB15 sample in 600 μL DMSO and plotting the integrals of the H_2O_2 peak versus time (Fig. S3). The kinetics of release are described by a sigmoidal curve, showing an initial lag phase (ca. 60 min), suggesting a non-labile interaction between the resin and hydrogen peroxide itself, other than a simple physisorption/absorption of H_2O_2 into the AMB15 inner network.

The multiple resonances observed for H_2O_2 NMR signal suggest the coexistence of multiple chemical environments for H_2O_2 in the presence of the resin (Fig S4A). In addition, the sharper H_2O_2 resonances, observed over AMB15 (H_2O_2 linewidth ~4 Hz), with respect to what observed in the absence of resin at similar concentrations (~15 Hz, Fig. 2B), points to a reduced chemical exchange of H_2O_2 protons with water in liquid phase, possibly related to H_2O_2 chemical interactions established with the chemical groups of the resin.

Altogether, NMR data support the hypothesis of H_2O_2 immobilization over the resin through non-labile chemisorption, such as the structures depicted in Scheme S1. NMR investigation is not able, however, to differentiate among various types of interactions.

1.3. Catalytic decontamination tests – An aliquot of 150 mg of resin were added to 10 mL of solvent (AcOEt, DCM, H_2O) containing 1745 ppm and 220 ppm, for CEES and malathion, respectively, in a batch glass reactor under stirring (600 rpm). The tests were performed in open air under mild conditions at 298 K and 1 bar pressure. The reaction advancement was monitored over time and the substrate conversion was computed via gas-chromatography with flame ionization detection (GC-FID, Agilent 6890).

A preliminary screening in CEES degradation showed AcOEt as the most promising reaction medium thanks to its good environmental sustainability, high performance, in terms of substrate conversion (Fig. S5), selectivity to desired products (cf. Fig. 1 and Fig. S6) and resin's recyclability (Fig. S16), and good solubility of all reactants. DCM, on the other hand, displayed good performance, but scarce sustainability as a chlorinated medium. At last, H_2O did not show interesting results, leading to a rapid deactivation of the abatement capability.

The experiments in anhydrous ethyl acetate, AcOEt, were performed in open air under mild conditions at 298 K and 1 bar pressure. Specifically, 150 mg of resin were added to 10 mL of substrate solution at 1745 ppm and 220 ppm, for CEES and malathion, respectively, in a batch glass reactor under stirring (600 rpm). The dispersion was stirred continuously throughout the test. Experiments with no solids (blank tests) were also carried out using the same procedure. The reactions were monitored over time and the corresponding degradation products were identified using various analytical techniques, including gas-chromatography with flame ionization detection (GC-FID, Agilent 6890), gas-chromatography-mass spectrometry (GC-MS, Agilent 8860 coupled to 5977C MSD), and NMR spectroscopy (Bruker Avance-400 MHz). All the above experiments were repeated three times for statistical purposes.
After the catalytic test, the spent resin was recovered, rinsed with ultrapure H_2O (MilliQ ultrapure water) and AcOEt, re-activated with aq. H_2O_2 and added to a fresh reaction batch.
Gas-chromatography (GC) was performed over an Agilent 6890 GC equipped with Flame-Ionization Detector (GC-FID) analysis (HP5, 30 m column; split mode).

1.4. Exchange with K_2CO_3 - The AMB15 resin was placed in a glass flask and stirred overnight with a 2 M K_2CO_3 aq. solution, followed by filtration and washing with deionized water until ca. pH 7 was reached. Dried in an oven for 30 min, the final resin was referred to as AMB15-K.

1.5. CHNS elemental analysis - Elemental analyses for C, H, N and S were conducted on AMB15 samples before and after the 4 catalytic cycles with resin recycling on a EuroVector EuroEA Elemental Analyser.

1.6. Bacterial inactivation tests – The antibacterial activity of resins (AMB15; AMB15+H_2O_2; AMB15-K; AMB15-K+H_2O_2) against *Escherichia coli* ATCC® 11229TM (Gram-) and *Staphylococcus aureus* ATCC® 6538TM b (Gram+) (Biogenetics Diagnostics srl) was evaluated following an in-house tested protocol [33, 34].
The bacteria were first inoculated onto Nutrient Agar using sterile applicator sticks provided by the manufacturer and incubated overnight at 37 °C. Subsequently, three individual colonies were collected using a sterile loop and transferred into bottles containing nutrient broth—peptone broth for *E. coli* and Brain Heart Infusion (BHI) broth for *S. aureus*. These cultures were incubated overnight at 37 °C.

An aliquot from the overnight culture was preserved in sterile Eppendorf tubes containing glycerol and diluted in Potassium Phosphate Buffer (KPi) in a 2:1:1 ratio. The tubes were sealed and stored at –80 °C. All bacterial cultures used for the antimicrobial activity tests were obtained from these frozen stocks.

The tests were performed using nutrient-rich media containing known concentration of bacteria in the range 10^9-10^{10} CFU/mL. Before testing, resins were sterilized by autoclaving at 121 °C, then treated with hydrogen peroxide. Resins were activated as described in NMR studies section. All tests were carried out in triplicate. Control experiments were also performed on resins not treated with H_2O_2.

Overnight bacterial cultures 10^9 CFU/mL were incubated with 200 mg of resin and samples were taken at 1 and 5 min. For cultures containing 10^{10} CFU/ml, an additional sample was collected after 10 min of contact. All the samples were then appropriately diluted, plated on agar, and incubated at 37 °C for 24 h. Samples activated with H_2O_2 were inactivated in the last step of dilution scheme with 0.3 μmol of catalase before plating them. All procedures were conducted under a laminar flow hood and each test was repeated on three resin samples. Surviving colonies were counted and the results were reported as Log(CFU) .

Instruments - UV lamp; pH meter; Autoclave AT80, TECNO-GAZ; Safe-mate Biological Safety Cabinet for bacteria class I and II; Incubator/refrigerated thermostat FALC mod. FTF180; Orbital shaker; Colony Counter 570, SUNTEX

1.7. Viral inactivation tests – All viral procedures were conducted in the Biosafety Level 3 Laboratory (BSL3) of Clinical Microbiology, Virology and Bioemergency of “Luigi Sacco” University Hospital (Milan, Italy).

HSV-1 and SARS-CoV-2 isolates were obtained from clinical samples and propagated in Vero E6 cells (ATCC® CRL-1586™). Vero E6 cells were maintained in Dulbecco’s Modified Eagle Medium with L-glutamine (DMEM, Gibco™ ThermoFisher Scientific), supplemented with 10% heat-inactivated fetal bovine serum (FBS, Gibco™ ThermoFisher Scientific) and 1% Penicillin-Streptomycin [5,000 U mL^{-1}] (Pen-Strep, Gibco™ ThermoFisher Scientific). Cultures were incubated at 37 °C and 5% CO_2 atmosphere.

Cells were routinely subcultured at 80–90% confluence and used for infection assays in multi-well plates according to the titration method.

For viral propagation, 1 mL of virus isolate was added to 9 mL of infection medium (DMEM supplemented with 2% FBS and 1% Pen-Strep), and flasks were incubated at 37 °C with 5% CO_2 for 72 h. The supernatant was centrifuged at 2000 × g for 5 min to remove cellular debris, aliquoted into cryotubes, and stored at -80 °C.

Virus stocks were thawed immediately before use and kept on ice during preparation of virucidal assays.

Virucidal activity was evaluated using AMB15, AMB15+H_2O_2, AMB15-K and AMB15-K+ H_2O_2 resins. For each experiment, 100 mg of resin were placed in sterile wells and exposed to 500 µL of virus suspension with known titre. Samples were incubated for the selected contact time (2 min for HSV-1 and SARS-CoV-2) at room temperature (Fig. S1).

After the contact time, the reaction was stopped by addition of sodium carbonate solution and catalase in potassium phosphate buffer in order to neutralize residual acidity and decompose hydrogen peroxide. The neutralized suspensions were immediately recovered from the resin phase and serially diluted in infection medium for subsequent infectivity assays. Cytotoxicity controls were performed in parallel by exposing confluent Vero E6 monolayers to treated resins and neutralization reagents under the same conditions used in virucidal assays.

Cell morphology was monitored microscopically after incubation and no cytopathic effect related to resin or neutralization reagents was observed. All inactivation experiments were carried out in triplicate. HSV-1 infectivity was assessed using a plaque reduction assay, with results expressed in PFU/mL (Plaque Forming Units per millilitre). This assay was performed for each experimental condition, and the results were compared to the positive control [35].
The assay was performed by infecting cell monolayers with serial dilutions of treated virus suspensions and counting plaques after incubation.
After virus adsorption, cells were overlaid with semi-solid medium and incubated until plaques were visible. Viral titres were calculated relative to untreated controls.

SARS-CoV-2 titre was determined by $TCID_{50}$ (Tissue Culture Infectious Dose 50%) assay in Vero E6 cells based on cytopathic effect evaluation and calculated according to the Spearman–Kärber method [36]. Serial dilutions of virus suspensions were added to cell cultures and incubated until cytopathic effects were observed.
The $TCID_{50}$ corresponded to the dilution producing infection in 50 % of wells.

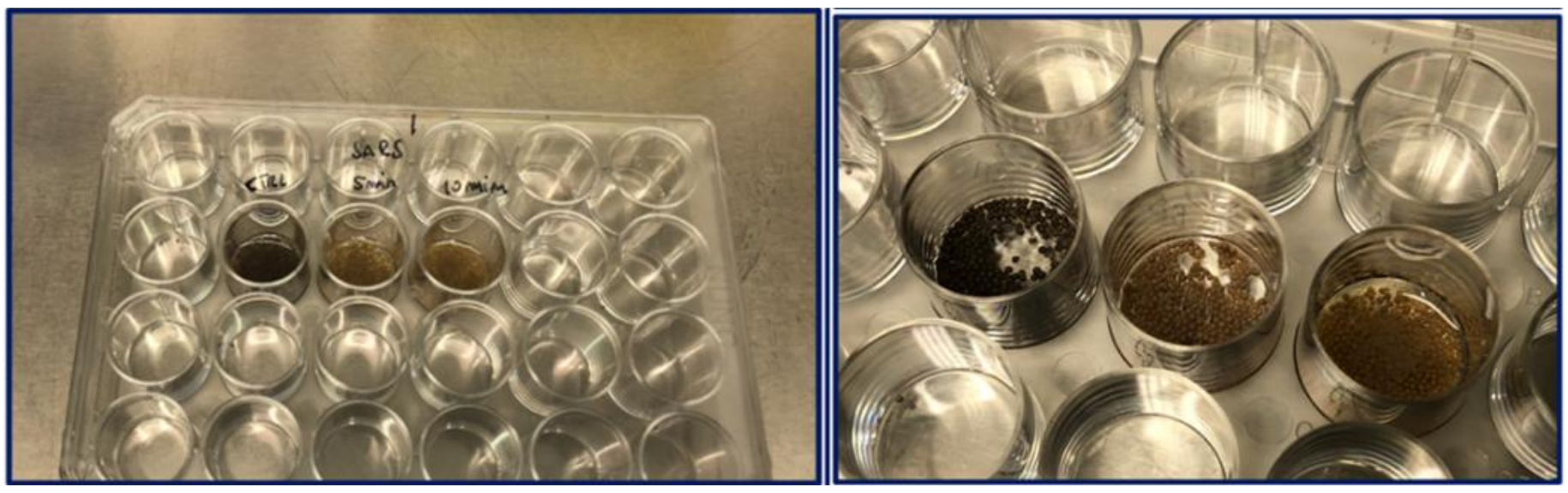

Figure S1. Virucidal assay performed in a 24-well plate. Different resins were dispensed into individual wells and exposed to virus suspensions for the selected contact time at room temperature. Each well contained 100 mg of resin and 500 µL of viral suspension.

2. Additional Information

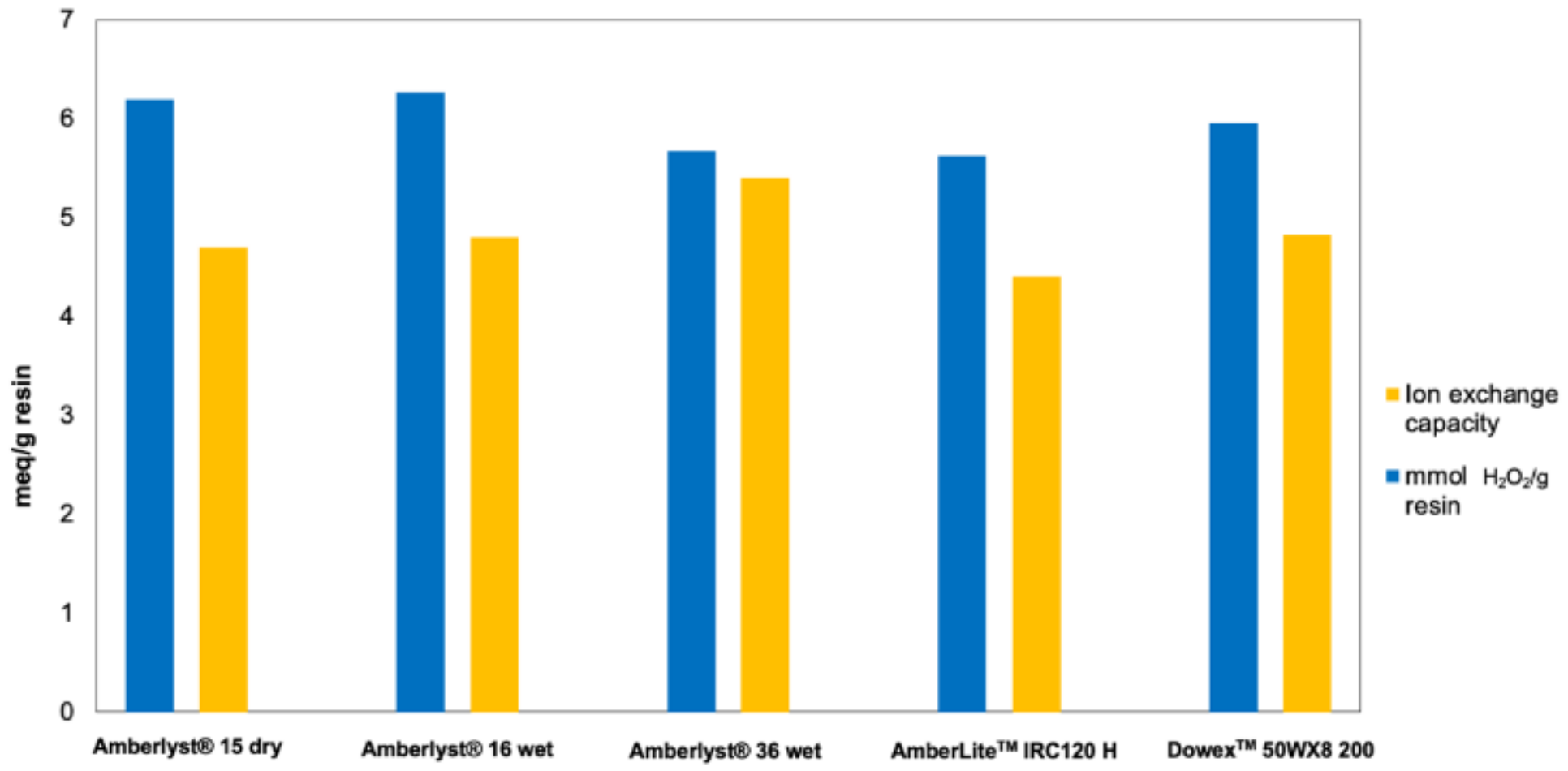

Figure S2. Density, expressed in $mmol_{H2O2}/g_{resin}$, of oxidant species (blue - experimentally calculated from data obtained by iodometric titrations) present on resins after treatment by immersion in 30 wt.% aq. H_2O_2 for 10 min, compared with ion exchange capacity (orange - data from manufacturer's technical data sheet).

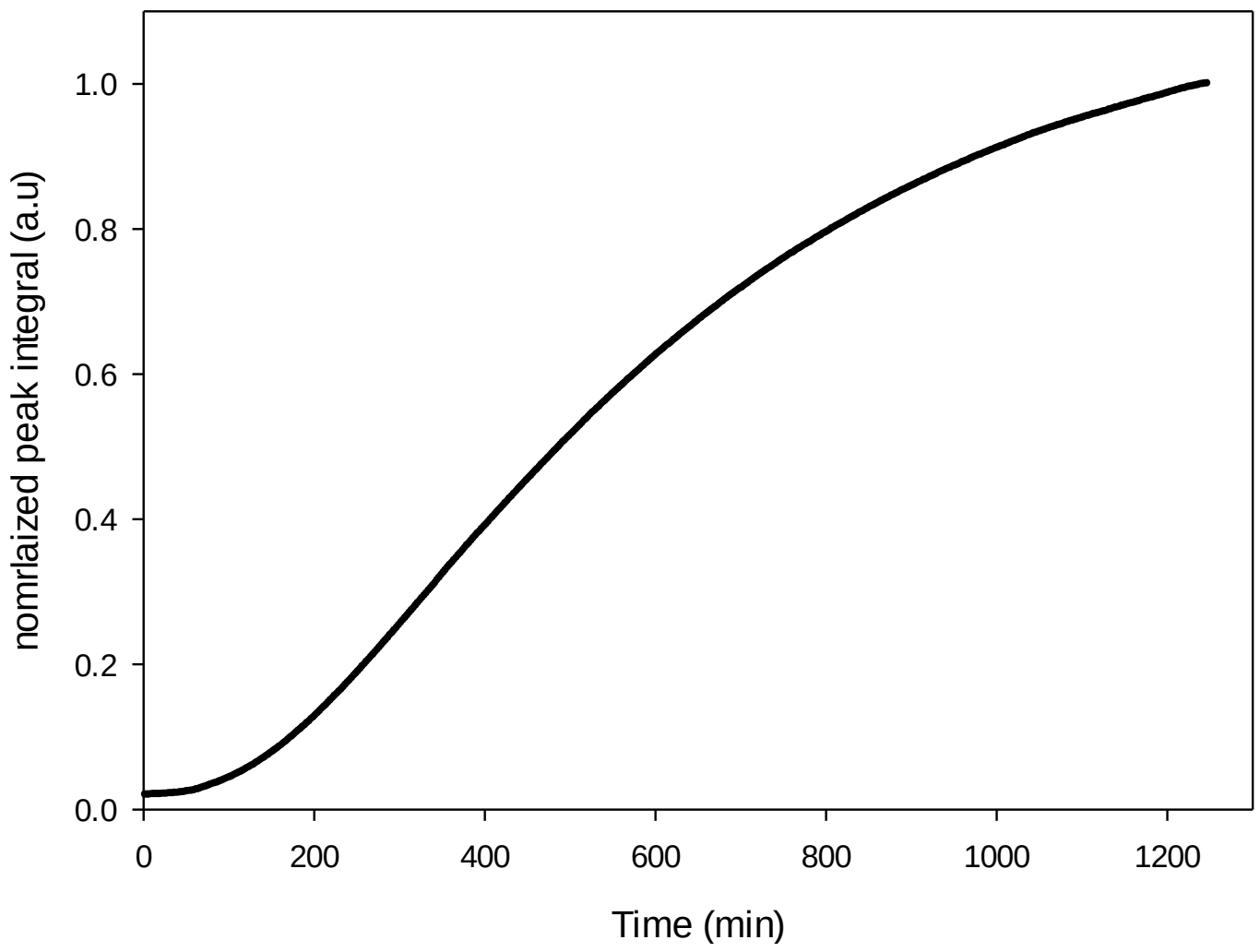

Figure S3. NMR-based kinetics of release of H_2O_2 (aq. 30 wt%) from AMB15. The curve is derived from the integration of H_2O_2 signals in 1D ^{1}H NMR spectra collected over time on a 10.8 mg AMB15 sample in 600 μL DMSO.

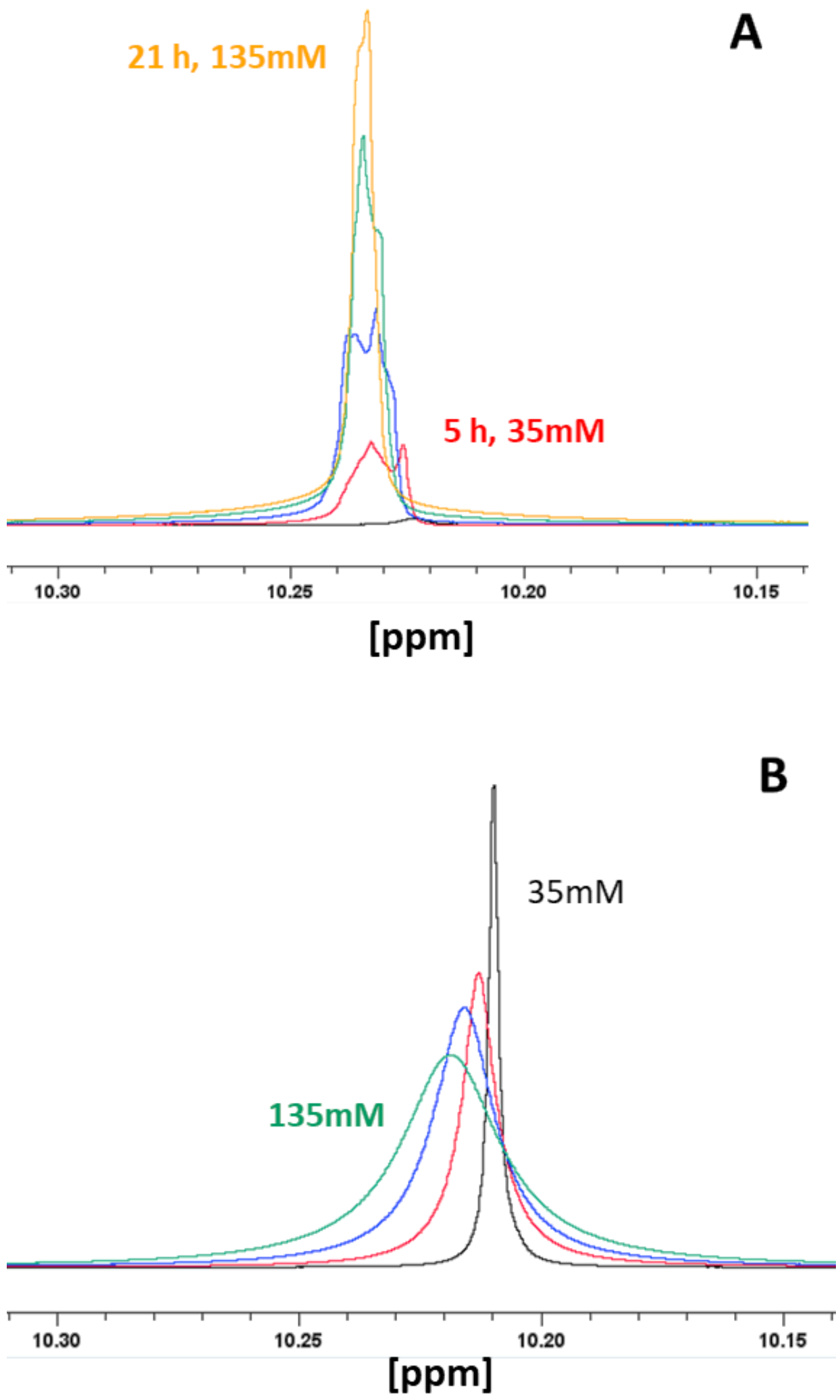

Figure S4. Solution NMR analysis of H_2O_2 resonances. (A) Overlay of 1D ^1H NMR spectra collected over time on a 10.8 mg AMB15 sample in 600 μL DMSO. The spectral region showing the H_2O_2 signals is shown. (B) Overlay of 1D ^1H NMR spectra of H_2O_2 (aq. 30 wt%) dissolved in DMSO at increasing concentrations (35, 66, 100, 135 mM). Spectra were collected at 500 MHz, 298 K.

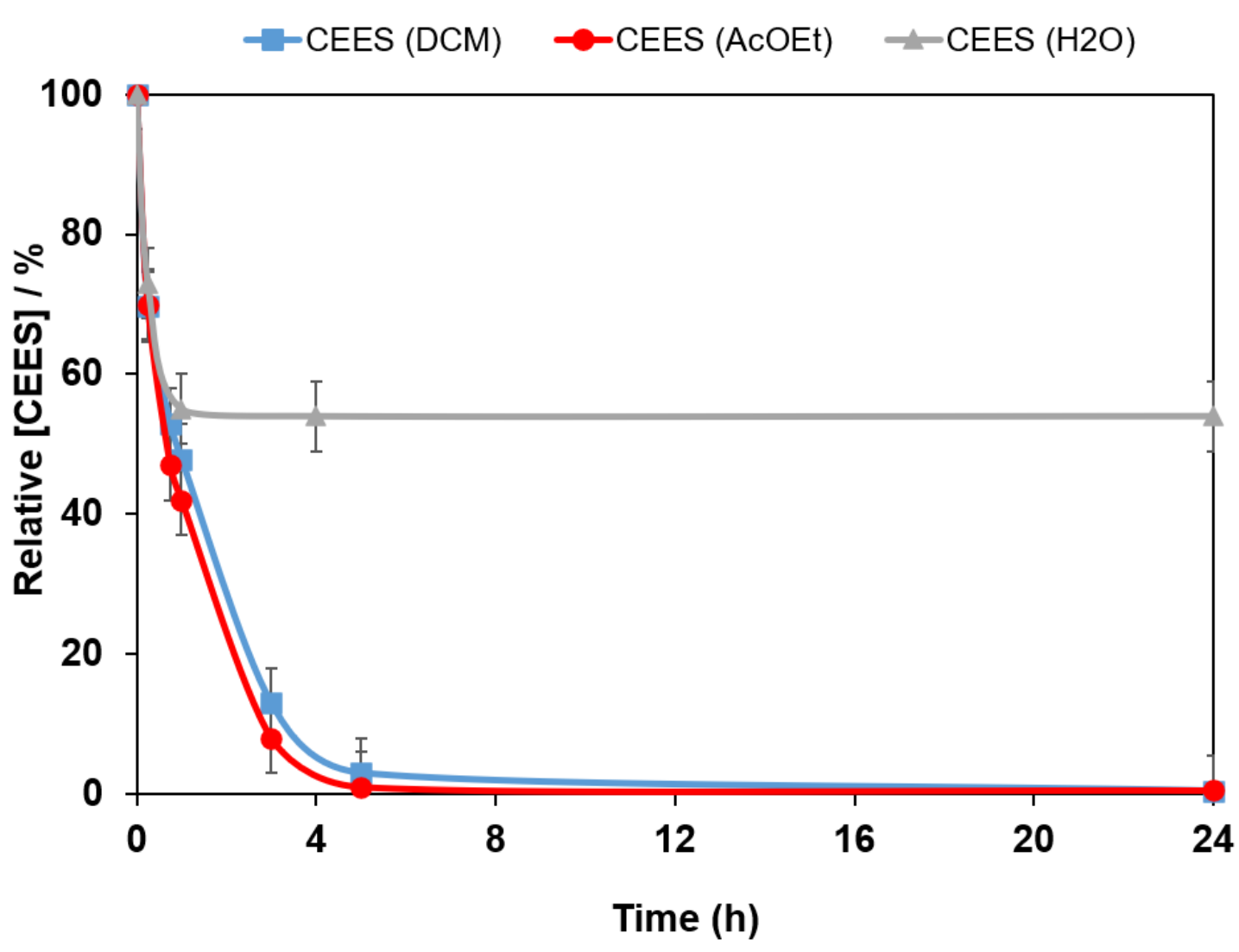

Figure S5. CEES degradation profiles over AMB15 in three different solvents: ethyl acetate (EtOAc), dichloromethane (DCM), and ultrapure water (H_2O). Reaction conditions: CEES concentration, 1745 ppm; 150 mg AMB15; CEES/H_2O_2 molar ratio, 1:5; 298 K; 1 bar. The residual CEES concentration was monitored by GC–FID and is expressed relative to its initial concentration.

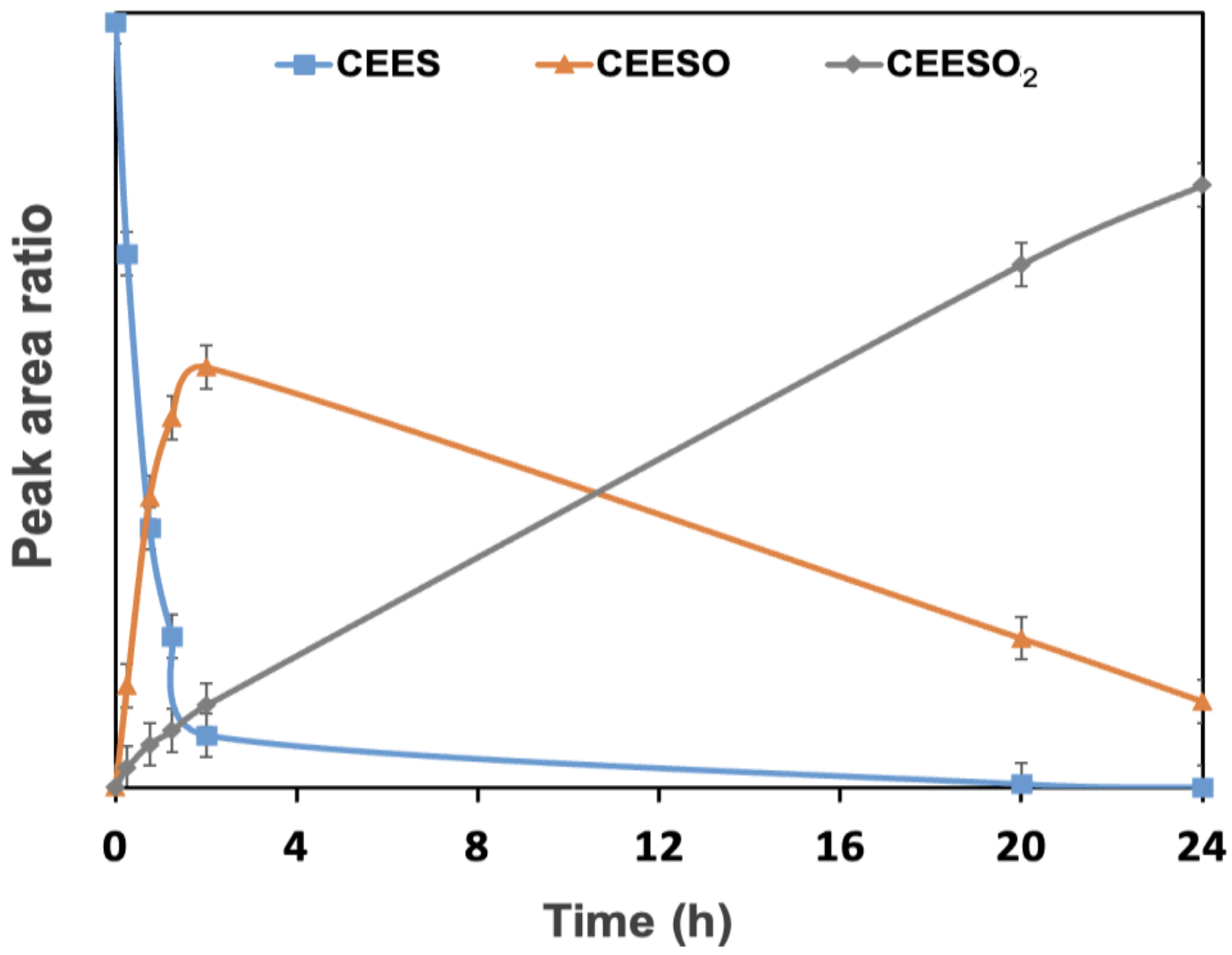

Figure S6. CEES degradation reaction profile over AMB15 and abundance of products formed (compound-to-internal std peak area ratio). Conditions: 1745 ppm CEES in DCM, 298 K, 1 bar, 150 mg cat; 1:5 CEES:H_2O_2 molar ratio, monitored by GC-FID. $EVSO_2$ profile not reported as its concentration is under detection limit.

¹H- and ¹³C-NMR characterisation of sulfur-containing compounds

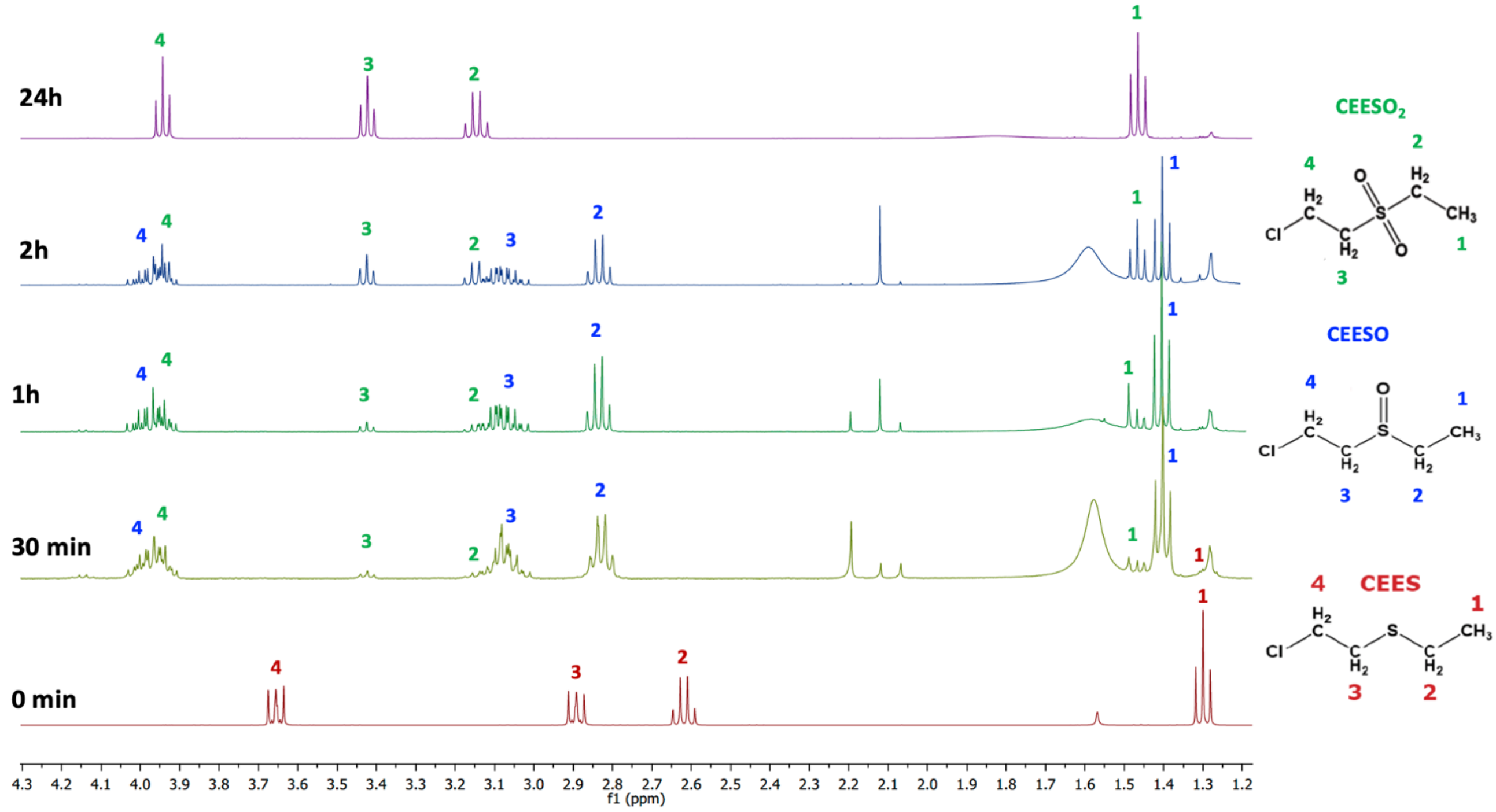

Figure S7. ^{1}H-NMR spectrum in $CDCl_3$ of CEES degradation reaction in presence of AMB15 and H_2O_2, monitored at different times (0 min, 30 min, 1 h, 2 h, 24 h). Reaction conditions: 10 mL CEES in AcOEt (14 mM), 298 K, 1 bar, 150 mg AMB15 + 72 μl H_2O_2 30 wt.%.

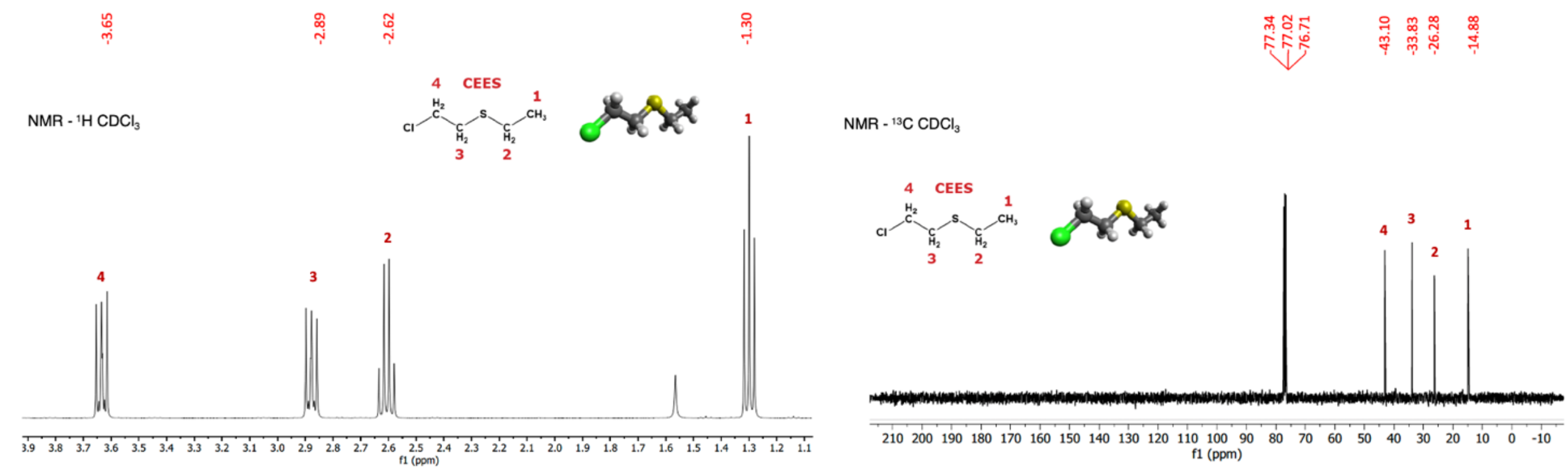

Figure S8. ^{1}H-NMR and ^{13}C-NMR spectrum in $CDCl_3$ of CEES

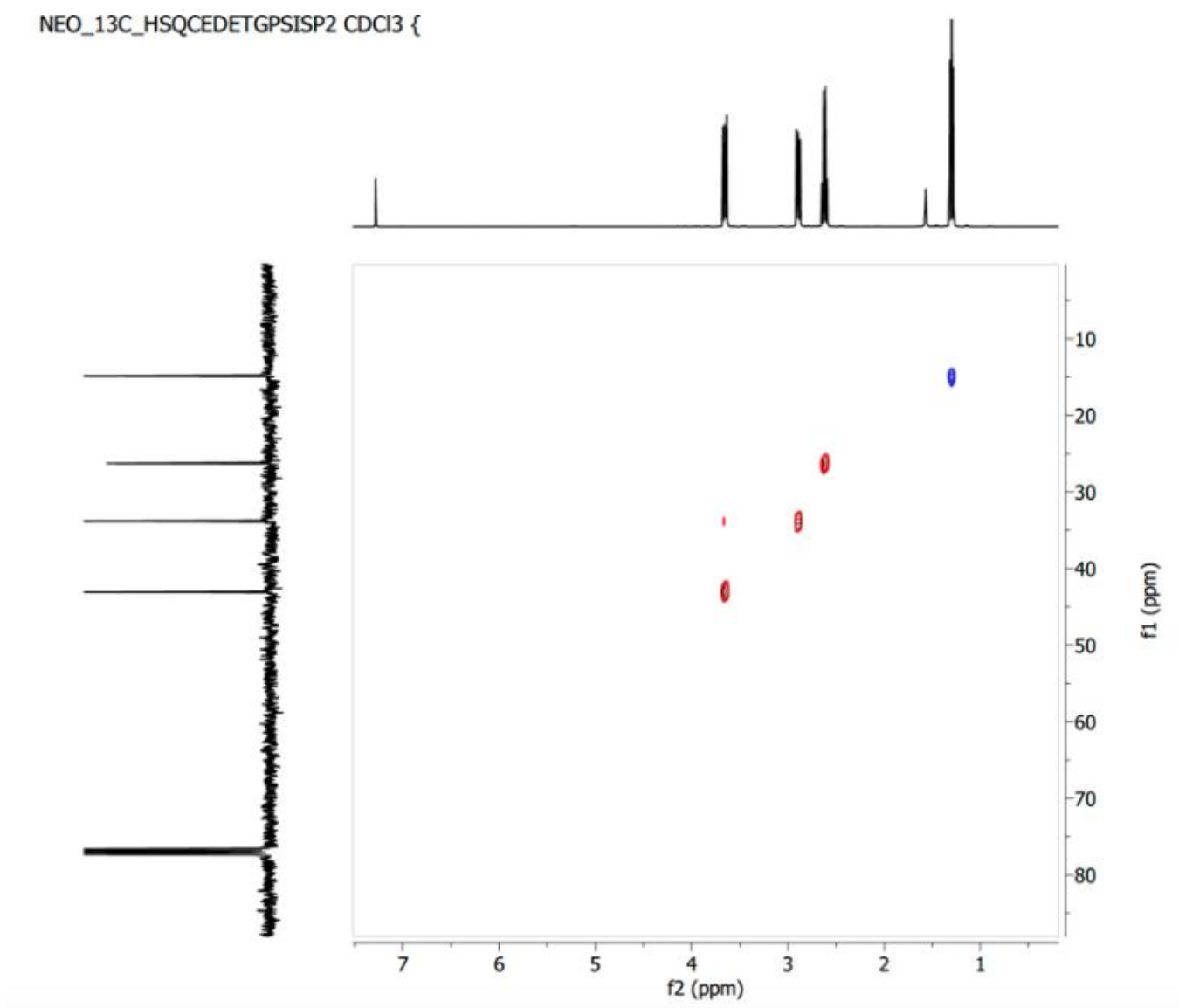

Figure S9. HSQC (Heteronuclear Single Quantum Coherence) - ^{13}C-NMR of CEES in $CDCl_3$

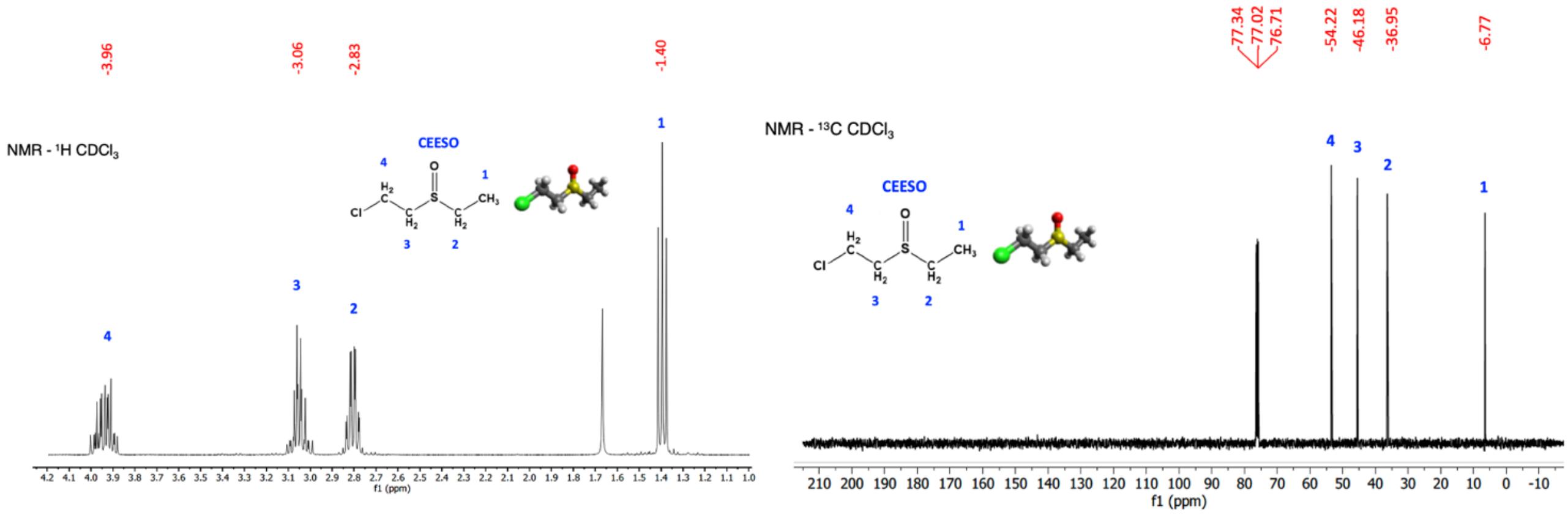

Figure S10. ^{1}H-NMR and ^{13}C-NMR spectrum in $CDCl_3$ of CEESO

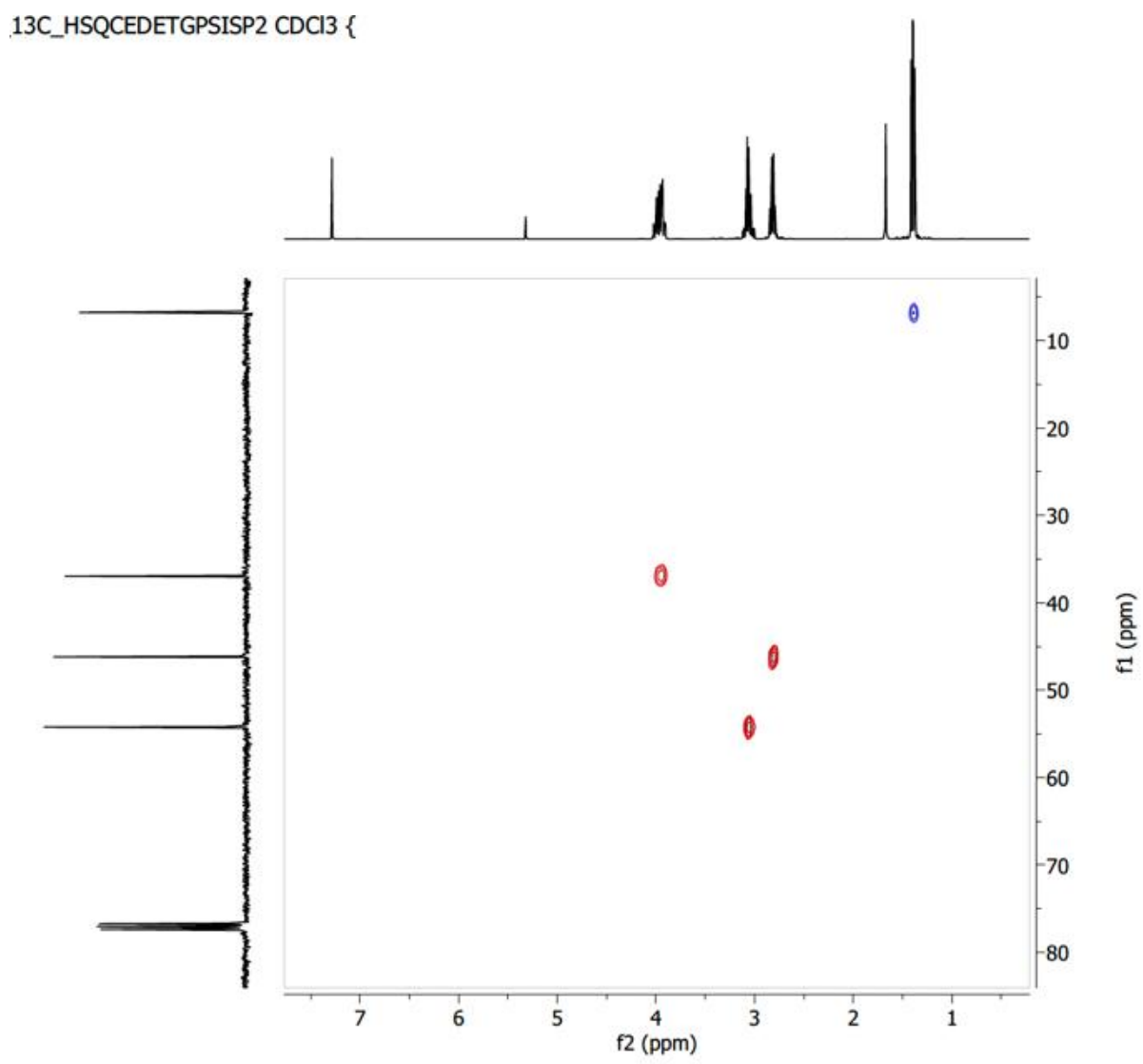

Figure S11. HSQC (Heteronuclear Single Quantum Coherence) - ^{13}C-NMR of CEESO in $CDCl_3$

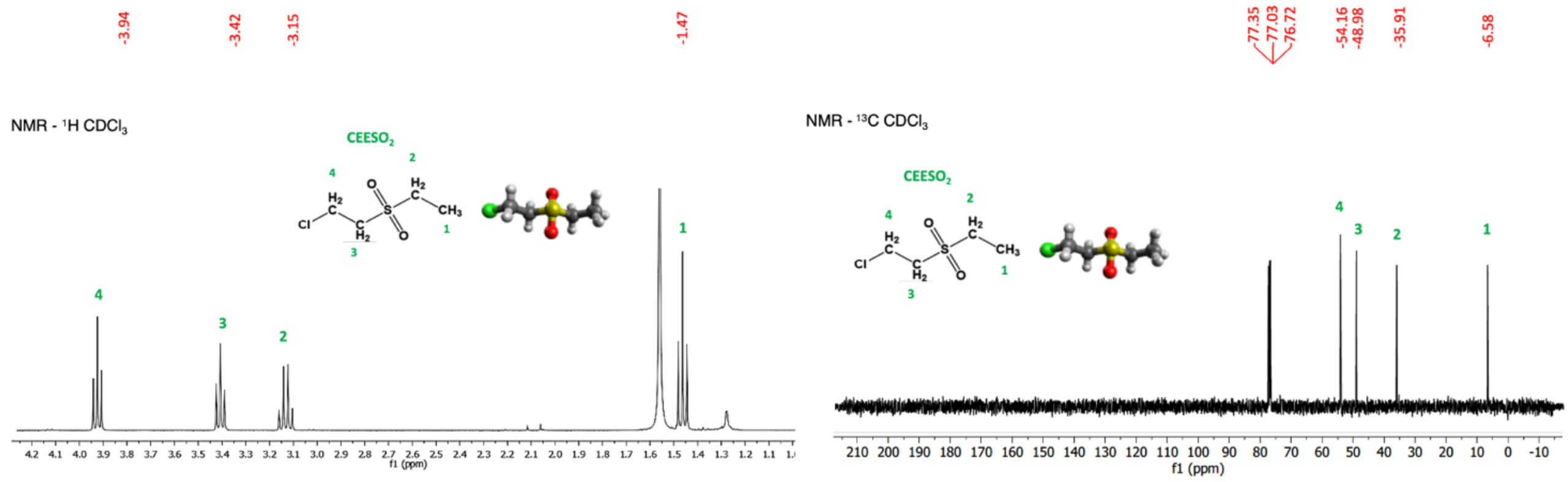

Figure S12. ^{1}H-NMR and ^{13}C-NMR spectrum in $CDCl_3$ of $CEESO_2$

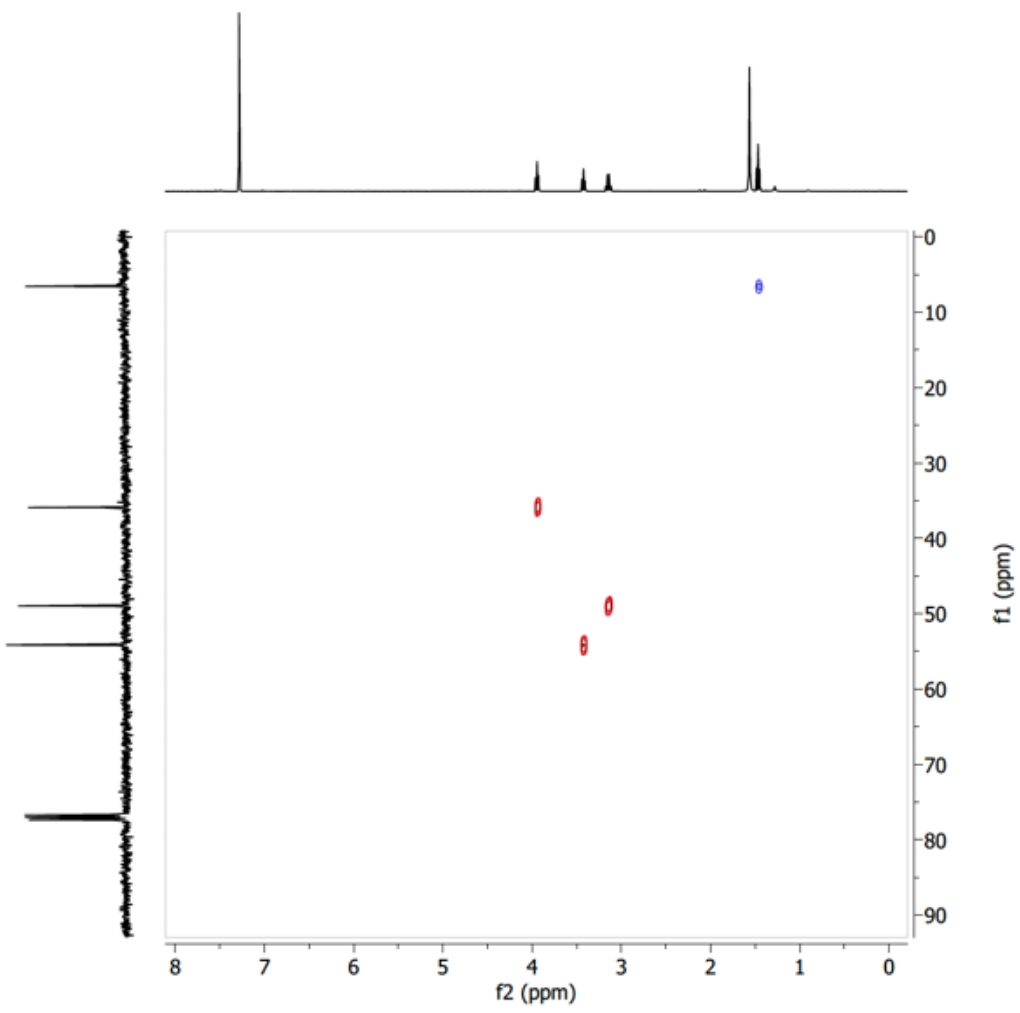

Figure S13. HSQC (Heteronuclear Single Quantum Coherence) - ^{13}C-NMR of $CEESO_2$ in $CDCl_3$

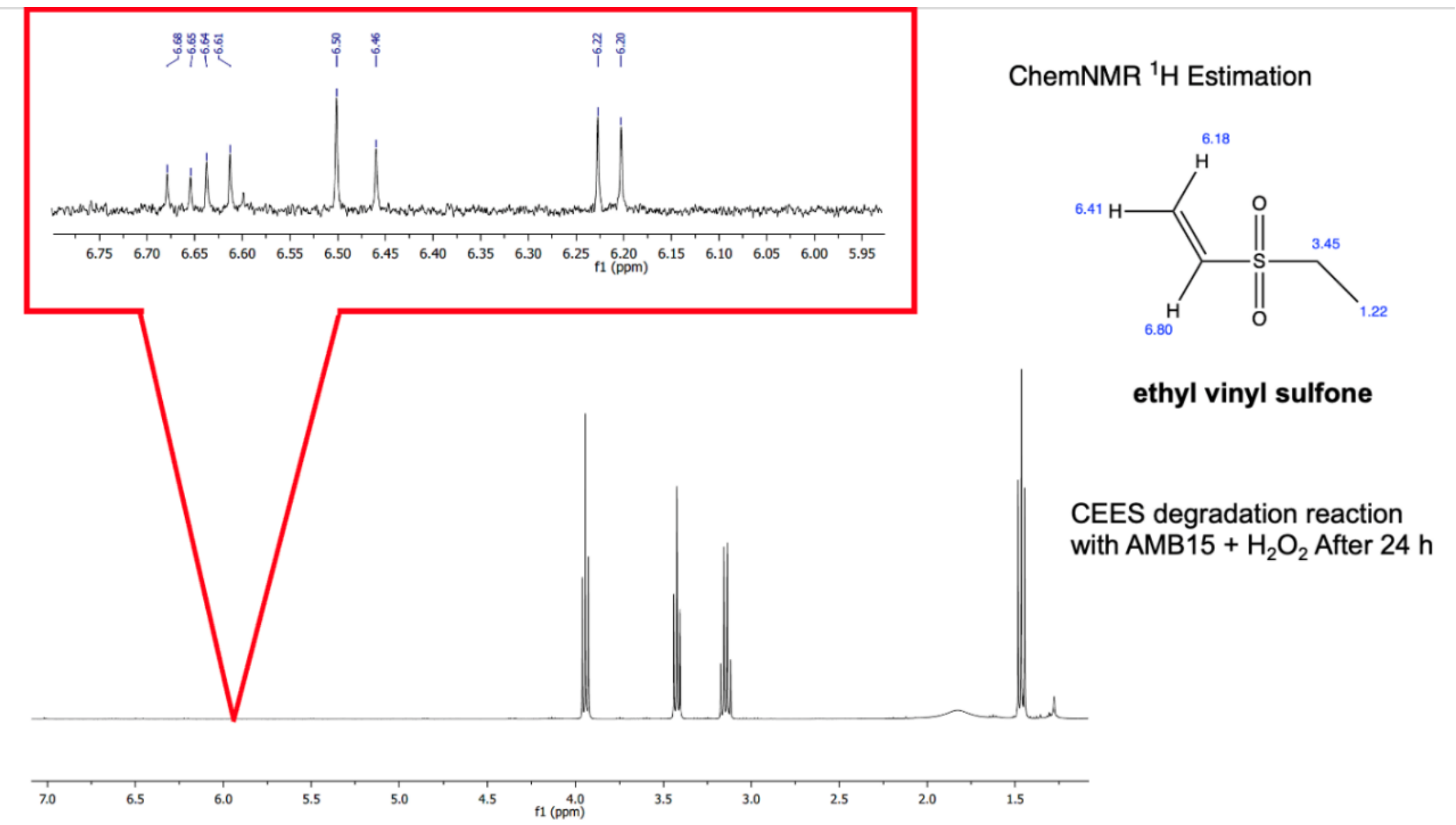

Figure S14. ^{1}H-NMR spectrum in $CDCl_3$ of CEES degradation reaction in presence of AMB15 and H_2O_2 after 24 h, with the detection of $EVSO_2$. Reaction conditions: 10 ml of CEES in AcOEt (14 mM), 298 K, 1 bar, 150 mg AMB15 + 72 μl H_2O_2 30 wt.%.

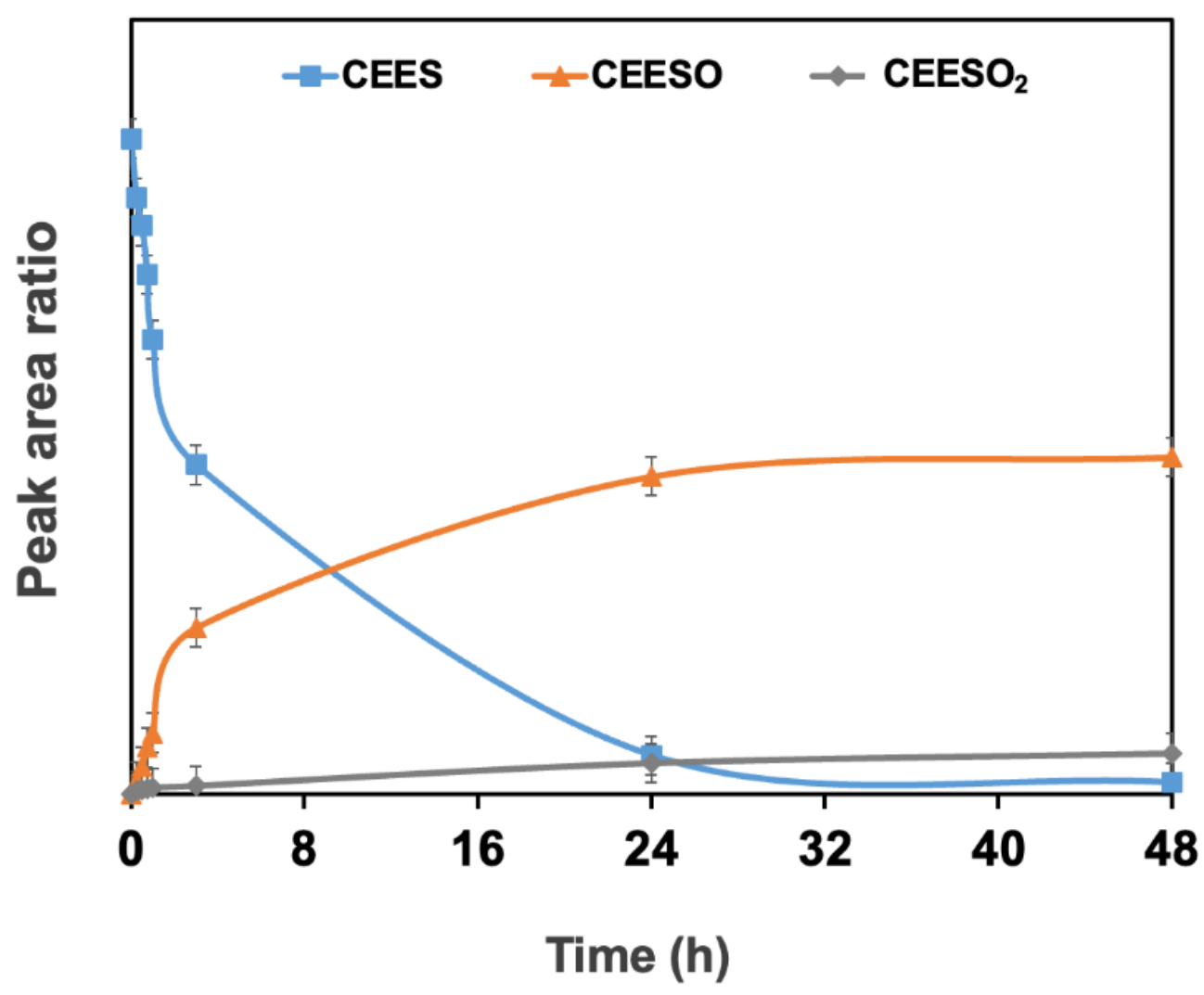

Figure S15. CEES degradation reaction profile over AMB15 and abundance of products formed (compound-to-internal std peak area ratio). Conditions: 1745 ppm CEES in AcOEt, 298 K, 1 bar, 150 mg resin; AMB15 resin treated with 6 wt.% aq. H_2O_2; products monitored by GC-FID.

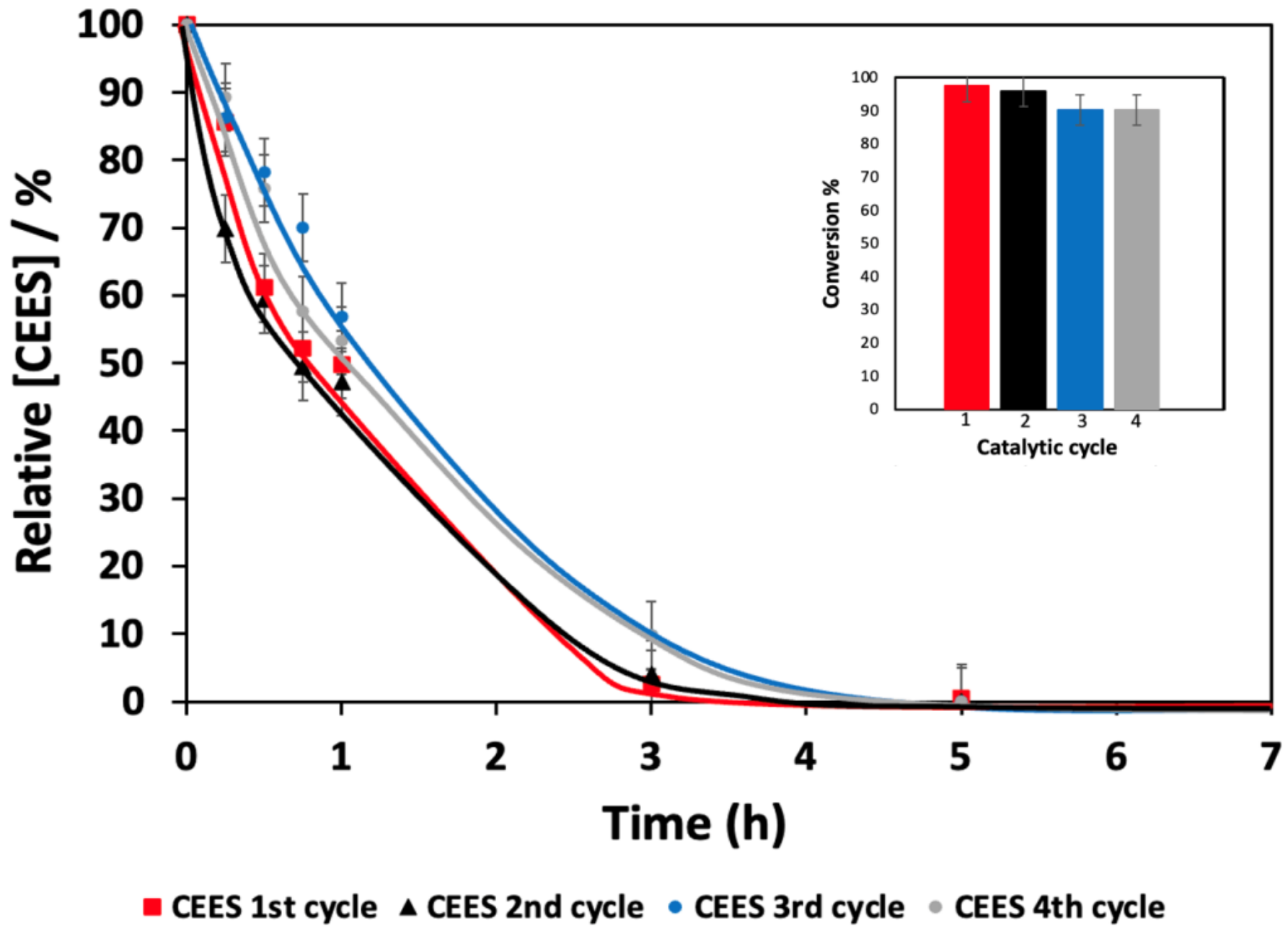

Figure S16. Concentration profile of CEES, monitored by GC-FID in the presence of AMB15 for the first (red line), second (black line), third (blue line) and fourth (grey line) catalytic abatement cycle. Reaction conditions for each test: 10 mL CEES in AcOEt (14 mM), 298 K, 1 bar, 150 mg AMB15 + 72 μL H_2O_2 30 wt.%. Inset: conversion values after 3 h of reaction.

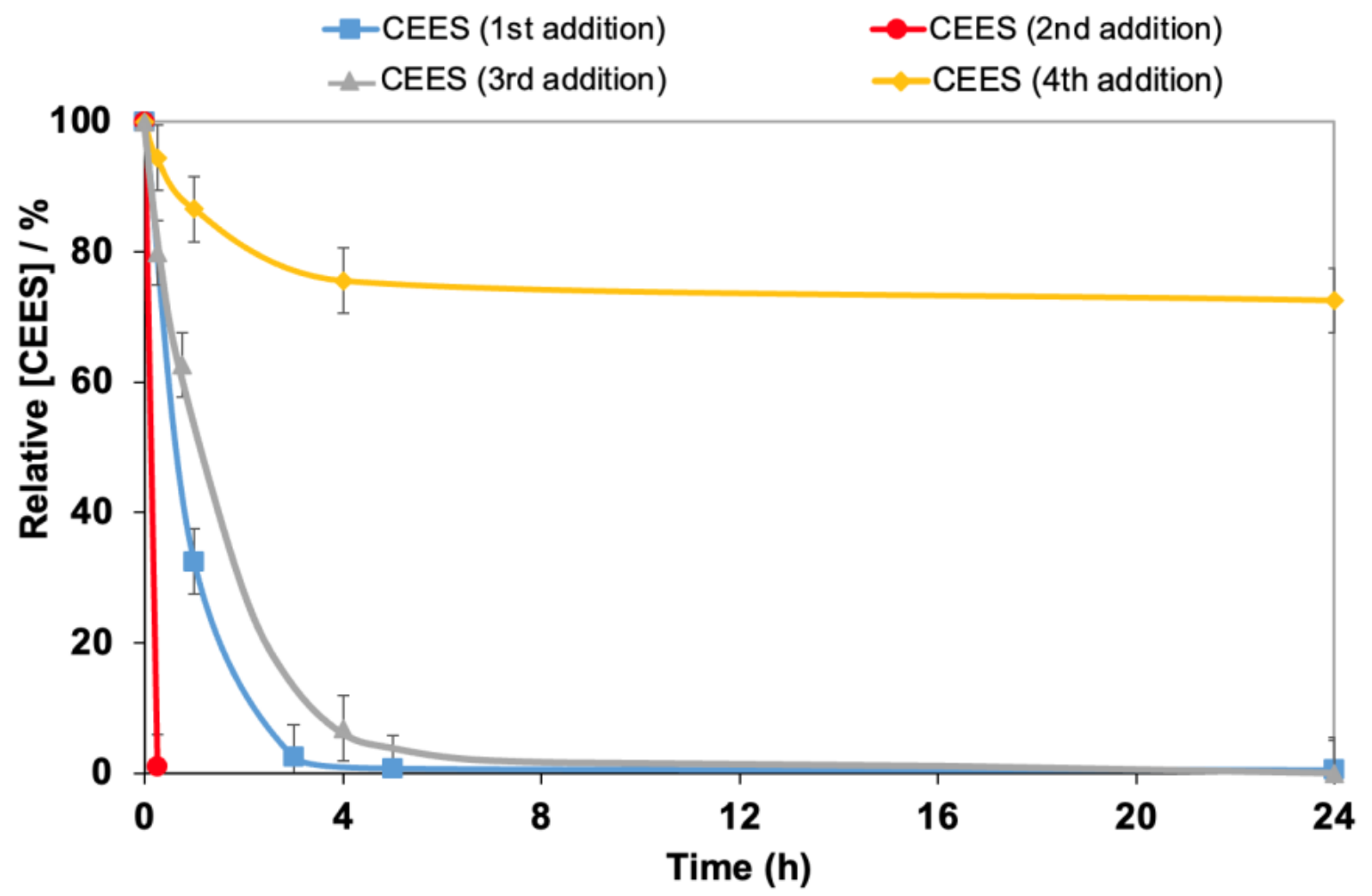

Figure S17. Comparison of reaction profiles for the four consecutive additions of aliquots of 16.4 µL of CEES (1745 ppm) in AcEOt, 298 K, 1 bar, 150 mg resin; 1:5 CEES:H_2O_2 molar ratio; monitored by GC-FID.

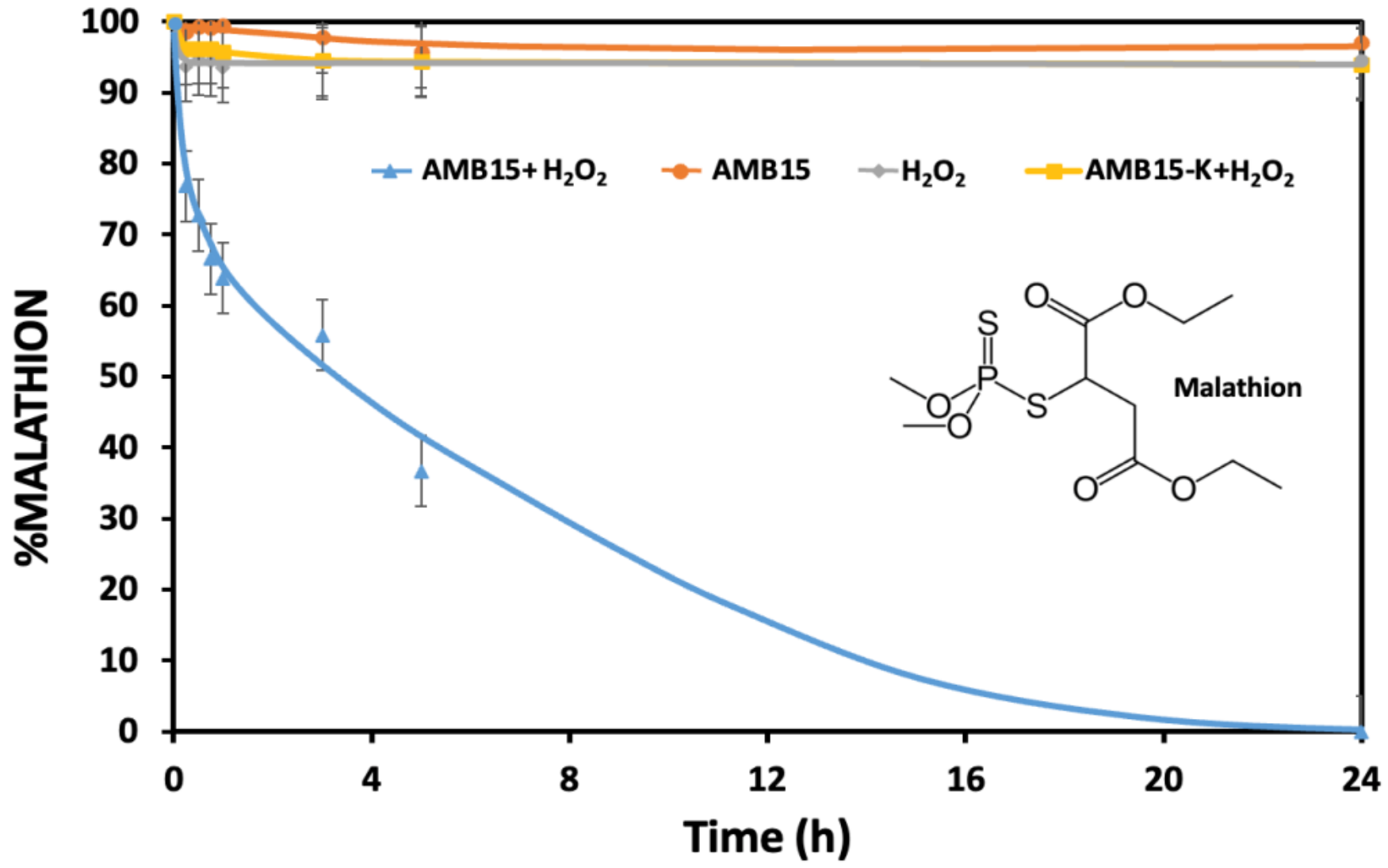

Figure S18. Malathion reaction profile with AMB15 + H_2O_2. Reaction conditions: 298 K, 1 bar, 150 mg AMB15, 10 ml Malathion 220 ppm (0.64 mM), 72 µL H_2O_2 30 wt.%.

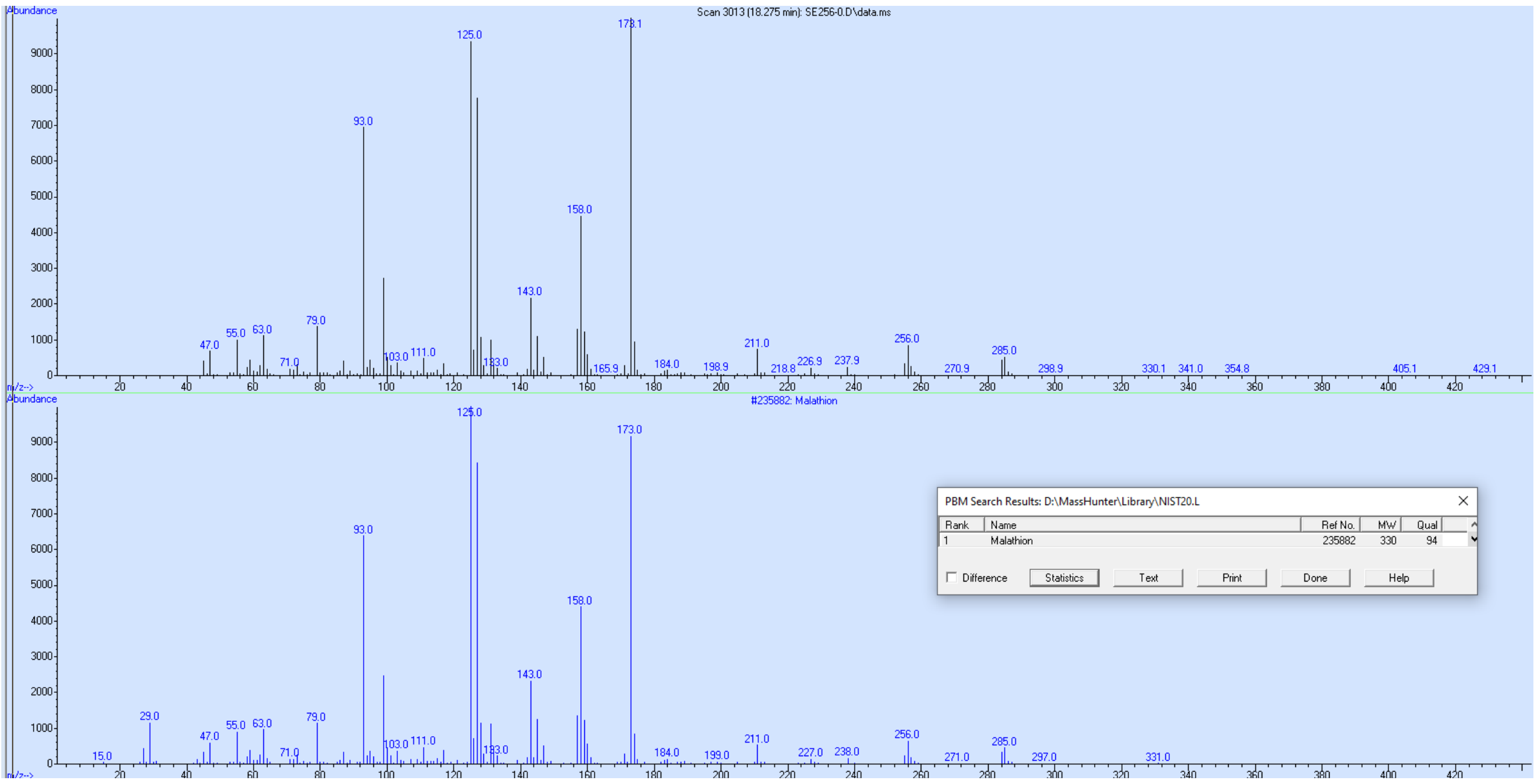

Figure S19. Mass spectra of diethyl malathion, collected at the maximum of the TIC chromatographic peak. Experimental recorded spectrum (above); reference spectrum (below).

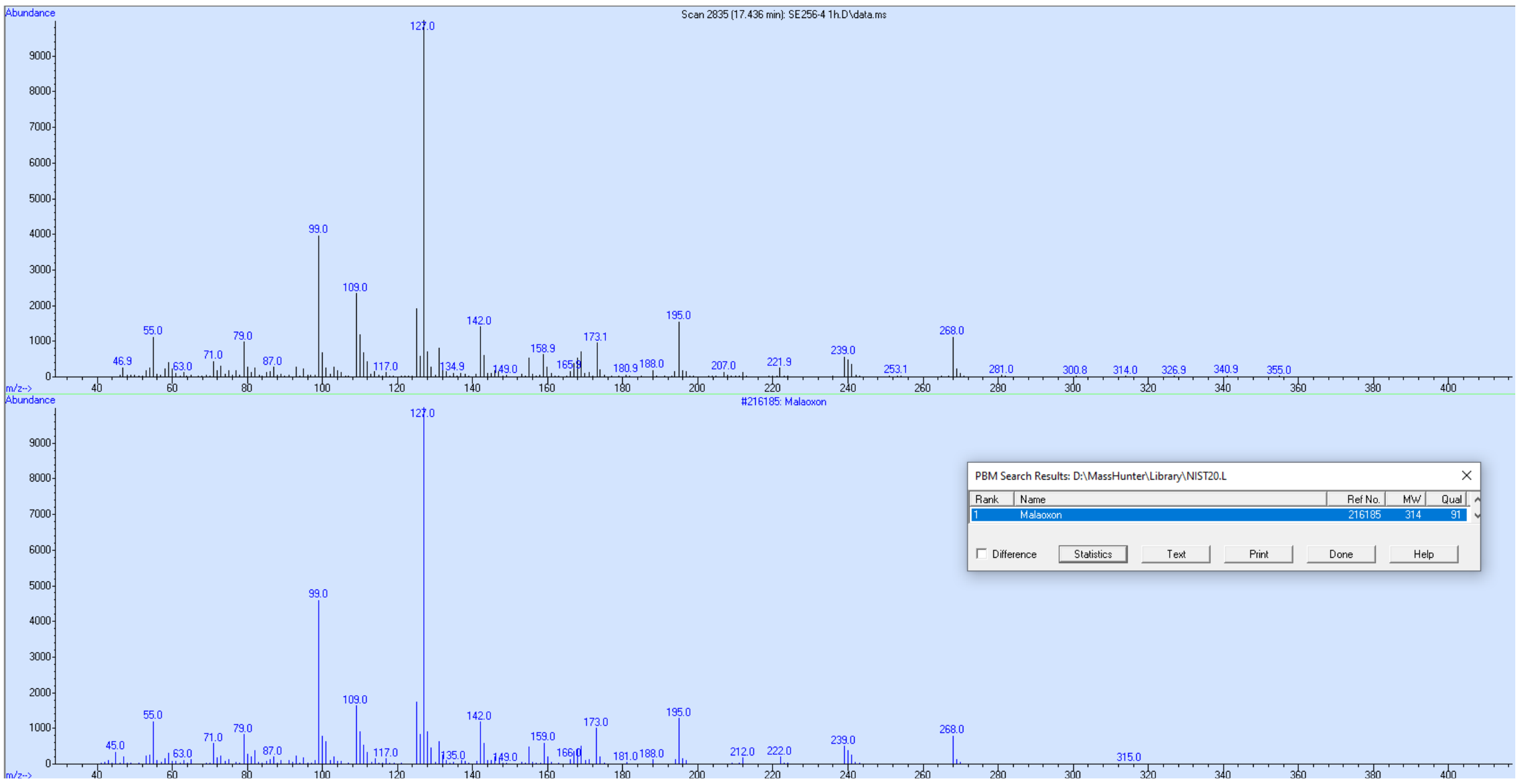

Figure S20. Mass spectra of malaoxon, collected at the maximum of the TIC chromatographic peak. Experimental recorded spectrum (above); reference spectrum (below).

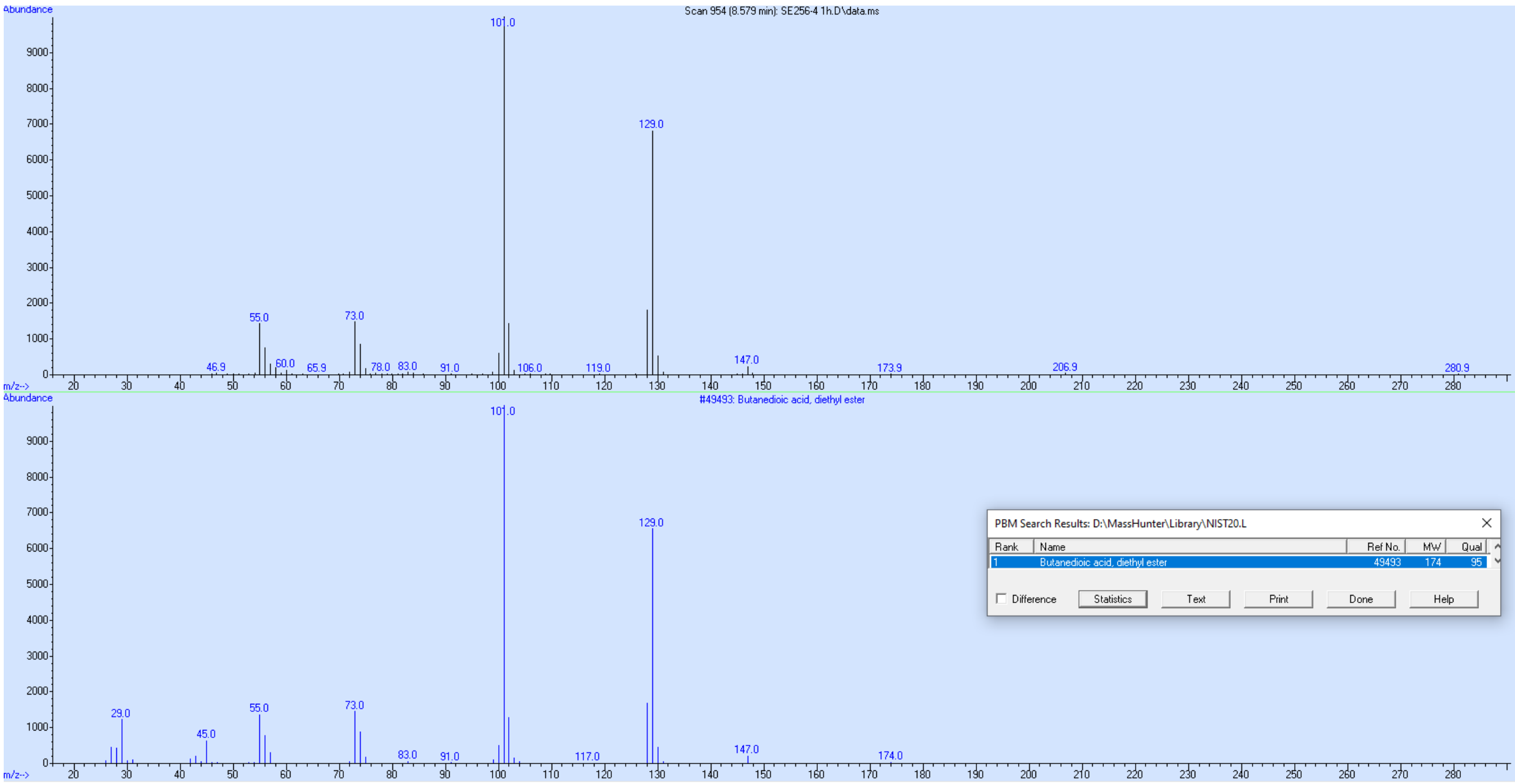

Figure S21. Mass spectra of diethyl succinate, collected at the maximum of the TIC chromatographic peaks. Experimental recorded spectrum (above); reference spectrum (below).

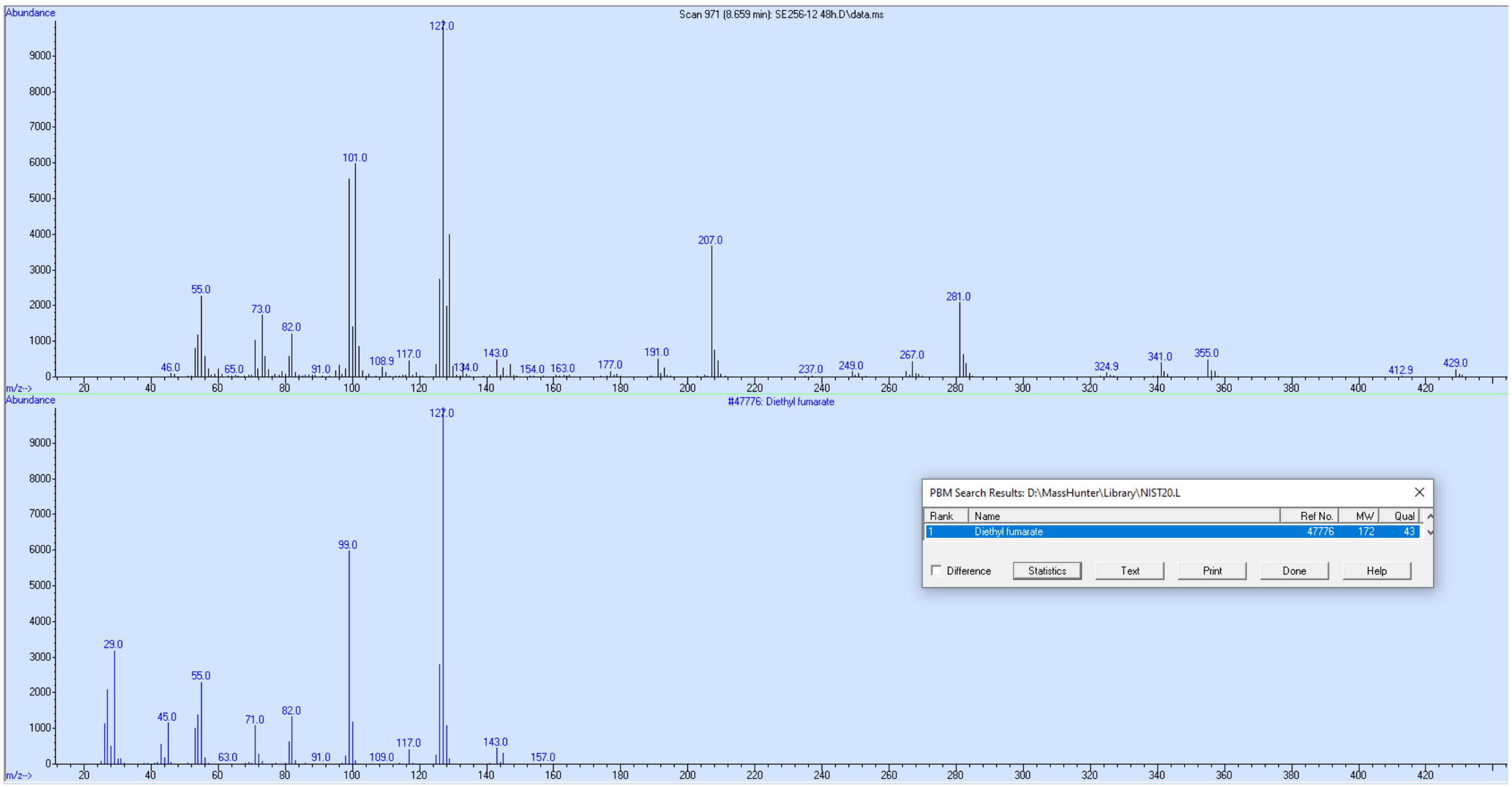

Figure S22. Mass spectra of diethyl fumarate, collected at the maximum of the TIC chromatographic peaks. Experimental recorded spectrum (above); reference spectrum (below).

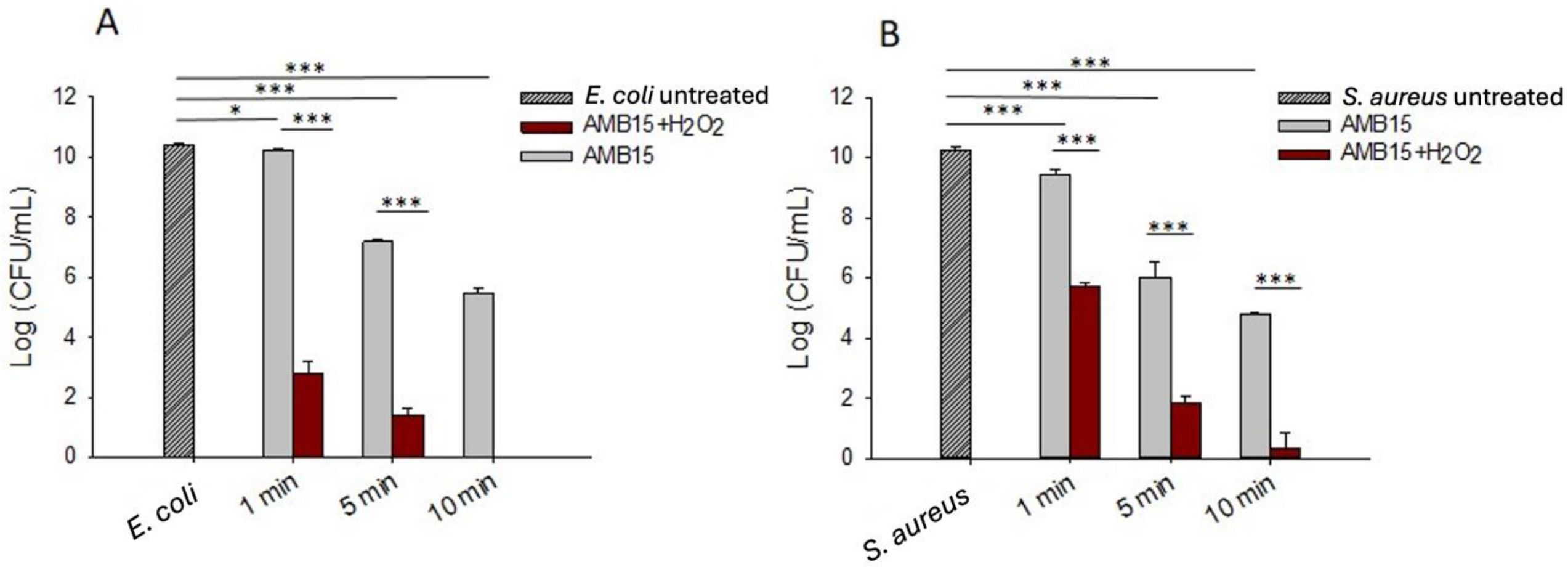

Figure S23. Antibacterial activity of the resins against *E. coli* (A) and *S. aureus* (B). Cell viability was measured for bacteria after exposure to AMB15 and AMB15-K resins, before and after H_2O_2 activation, at different contact times (min). Data were collected on starting bacteria cultures of 10^{10} CFU/ml and presented in logarithmic scale as average of three independent measurements. In panel A, no surviving cells were detected after 10 min of contact with AMB15 + H_2O_2. Differences between treated samples and untreated *E. coli* or *S. aureus* were evaluated using two-tailed Student's t-test; *, p< 0.05; **, p<0.01; ***, p<0.001.

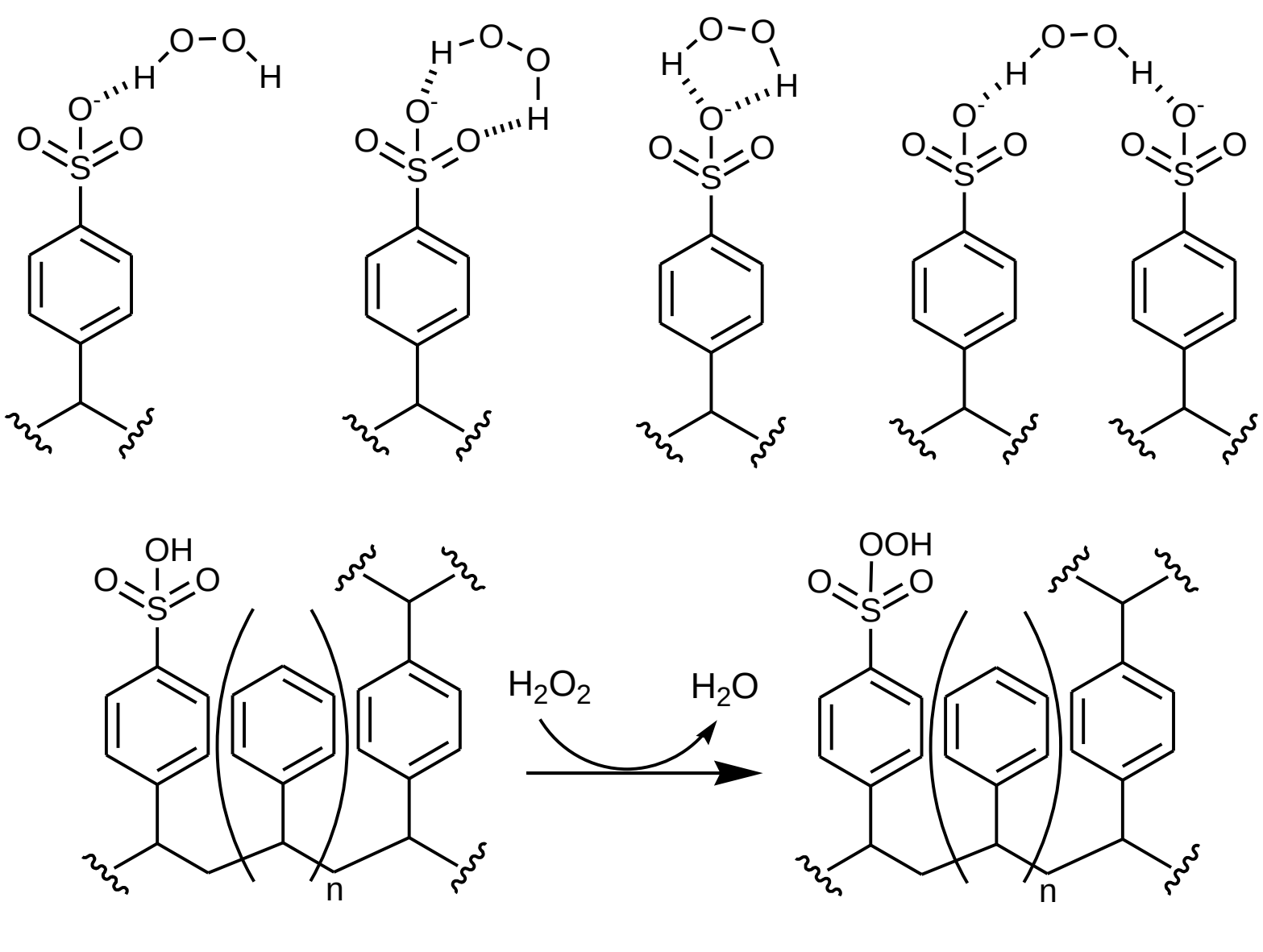

Scheme S1. Possible immobilised oxidizing species obtained via chemisorption or hydrogen-bond interaction between the arenesulfonic styrene-divinylbenezene ion-exchange resin and H_2O_2

Table S1. Amount of oxidant species calculated experimentally through iodometric titrations and the ion exchange capacity of each resin obtained from manufacturer's technical data sheet.

Resin	Ion Exchange Capacity (meq/g by dry weight) [a]	Oxidant species (mmol H_2O_2/g resin) [b]
**Amberlyst® 15 dry**	4.7	6.19
**Amberlyst® 16 wet**	4.8	6.27
**Amberlyst® 36 wet**	>5.40	5.67
**AmberLite™ IRC120 H**	4.4	5.62
**Dowex™ 50WX8 200**	4.83	5.95

[a] Data from technical data sheet. [b] experimental data obtained by iodometric titrations.

Table S2. Comparison of activity (% CEES conversion) of the five tested resins at different times.

	% CEES conversion after			
Resin	0.25 h	1 h	3 h	24 h
**Amberlyst® 15 dry**	20.6	51.2	88.2	>99.5
**Amberlyst® 16 wet**	2.5	22.9	67.4	>99.5
**Amberlyst® 36 wet**	28.9	46.8	83.6	>99.5
**AmberLite™ IRC120 H**	8.9	18.7	34.1	>99.5
**Dowex™ 50WX8 200**	8.0	17.3	41.8	>99.5
**AMB15-K + H_2O_2**	0	2.9	2.9	4.4
**Resin blank (with AMB15)**	0	0	1.5	7.0
**H_2O_2 blank**	0	0.5	0.5	0.9

Reaction conditions: resin previously activated with aq. H_2O_2 in contact with 10 mL of CEES in AcOEt (14 mM) (molar ratio H_2O_2/CEES 5:1), 298 K, 1 bar

Table S3. CHNS elemental analyses (wt.%) performed on AMB15 samples before and after 4 catalytic cycles.

#	Type	Name	N %	C %	H %	S %	O %	Weight (mg)
1	Byp	Bypass	-	-	-	-	-	-
2	Blk	Blank	-	-	-	-	-	-
3	Std	Sulphanilamide	16.261	41.838	4.452	18.568	-	0.543
4	Std	Sulphanilamide	16.273	41.858	4.988	18.676	-	1.168
5	Std	Sulphanilamide	16.265	41.844	4.546	18.600	-	1.689
6	Blk	Blank	-	-	-	-	-	-
7	Smp	AMB 15_after 4 catalytic cycle	0.179	41.034	6.820	9.629	-	1.003
8	Smp	AMB 15_after 4 catalytic cycle	0.155	40.922	5.885	10.544	-	1.648
9	Smp	AMB 15_after 4 catalytic cycle	0.151	41.110	5.911	9.500	-	1.576
10	Smp	AMB 15	0.108	42.102	6.003	13.535	-	1.065
11	Smp	AMB 15	0.076	41.248	5.972	12.205	-	1.518

Table S4. Malathion and main degradation products

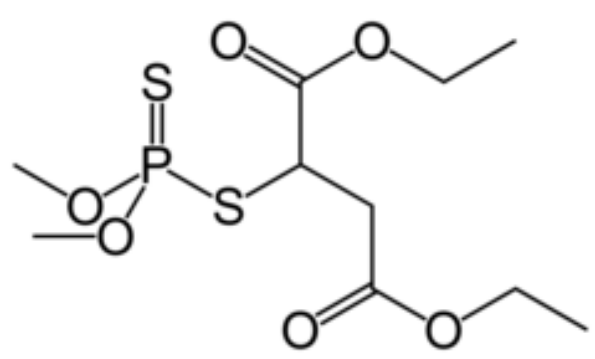 malathion diethyl<br>MW = 330.35 g mol^{-1}<br>retention time = 18.1 min[a]	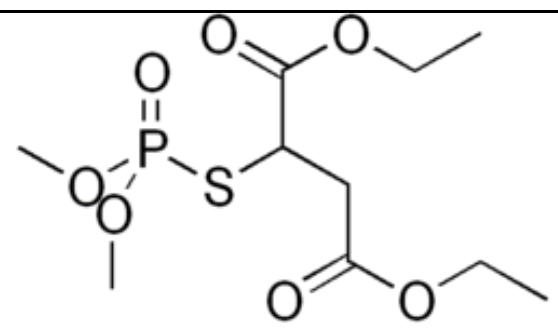 malaoxon diethyl<br>MW = 314.29 g mol^{-1}<br>retention time = 17.4 min[a]
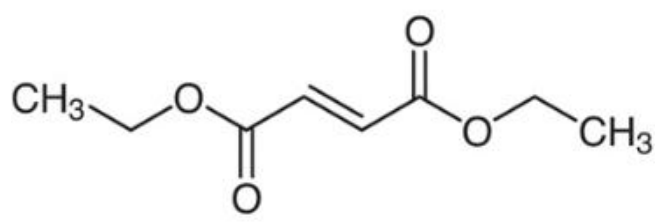 diethyl fumarate<br>MW = 172.18 g mol^{-1}<br>retention time = 8.65 min[a]	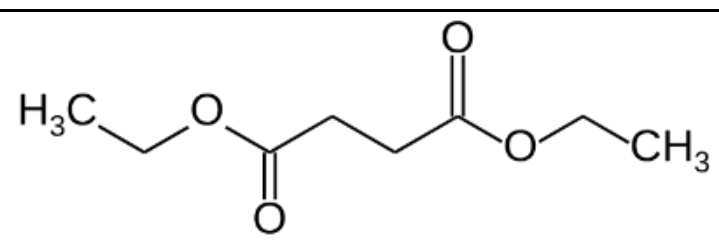 diethyl succinate<br>MW = 174.19 g mol^{-1}<br>retention time = 8.55 min[a]

[a] GC-MS retention time (GC-MS; HP-5ms UI, 30 m, 0.25 mm ø, 0.25 μm; temperature programme: initial T 60°C, 2 min, 10°C/min to 290°C, 4 min) as recorded under the analysis conditions described
